# Machine learning-based risk predictive models for diabetic kidney disease in type 2 diabetes mellitus patients: a systematic review and meta-analysis

**DOI:** 10.3389/fendo.2025.1495306

**Published:** 2025-03-03

**Authors:** Yihan Li, Nan Jin, Qiuzhong Zhan, Yue Huang, Aochuan Sun, Fen Yin, Zhuangzhuang Li, Jiayu Hu, Zhengtang Liu

**Affiliations:** ^1^ Department of Geriatrics, Xiyuan Hospital, China Academy of Traditional Chinese Medicine, Beijing, China; ^2^ Faculty of Chinese Medicine, Macau University of Science and Technology, Macao, Macao SAR, China; ^3^ Graduate School of Beijing University of Chinese Medicine, Beijing, China

**Keywords:** machine learning, predictive model, type 2 diabetes mellitus, diabetic kidney disease, meta-analysis, systematic review

## Abstract

**Background:**

Machine learning (ML) models are being increasingly employed to predict the risk of developing and progressing diabetic kidney disease (DKD) in patients with type 2 diabetes mellitus (T2DM). However, the performance of these models still varies, which limits their widespread adoption and practical application. Therefore, we conducted a systematic review and meta-analysis to summarize and evaluate the performance and clinical applicability of these risk predictive models and to identify key research gaps.

**Methods:**

We conducted a systematic review and meta-analysis to compare the performance of ML predictive models. We searched PubMed, Embase, the Cochrane Library, and Web of Science for English-language studies using ML algorithms to predict the risk of DKD in patients with T2DM, covering the period from database inception to April 18, 2024. The primary performance metric for the models was the area under the receiver operating characteristic curve (AUC) with a 95% confidence interval (CI). The risk of bias was assessed using the Prediction Model Risk of Bias Assessment Tool (PROBAST) checklist.

**Results:**

26 studies that met the eligibility criteria were included into the meta-analysis. 25 studies performed internal validation, but only 8 studies conducted external validation. A total of 94 ML models were developed, with 81 models evaluated in the internal validation sets and 13 in the external validation sets. The pooled AUC was 0.839 (95% CI 0.787-0.890) in the internal validation and 0.830 (95% CI 0.784-0.877) in the external validation sets. Subgroup analysis based on the type of ML showed that the pooled AUC for traditional regression ML was 0.797 (95% CI 0.777-0.816), for ML was 0.811 (95% CI 0.785-0.836), and for deep learning was 0.863 (95% CI 0.825-0.900). A total of 26 ML models were included, and the AUCs of models that were used three or more times were pooled. Among them, the random forest (RF) models demonstrated the best performance with a pooled AUC of 0.848 (95% CI 0.785-0.911).

**Conclusion:**

This meta-analysis demonstrates that ML exhibit high performance in predicting DKD risk in T2DM patients. However, challenges related to data bias during model development and validation still need to be addressed. Future research should focus on enhancing data transparency and standardization, as well as validating these models’ generalizability through multicenter studies.

**Systematic Review Registration:**

https://inplasy.com/inplasy-2024-9-0038/, identifier INPLASY202490038.

## 1 Introduction

Diabetic kidney disease (DKD) is one of the major microvascular complications of type 2 diabetes mellitus (T2DM), characterized by both structural and functional renal impairment resulting from chronic hyperglycemia. The global incidence of T2DM has shown a marked increase, which has been paralleled by a rising prevalence of DKD (1, 2). It is estimated that approximately 20% to 40% of individuals with diabetes will eventually develop DKD (1, 3). DKD remains a leading cause of end-stage renal disease (ESRD) worldwide (4–6). The presence and severity of DKD have been demonstrated to significantly elevate the risk of adverse health outcomes, including premature mortality, in patients with T2DM (7). This poses a substantial challenge to global public health. Consequently, the early identification of individuals at high risk for DKD is critical for implementing targeted interventions and improving clinical prognosis.

Conventional risk prediction models for DKD, such as linear regression, estimate risk by calculating a weighted sum of known risk factors (8–10). While these models are effective when a limited number of variables or predictors are involved, they face significant limitations in capturing the complex nonlinear relationships and interactions among multidimensional risk factors (11, 12). In contrast, ML methods that leverage big data or multidimensional datasets offer substantial promise in improving predictive accuracy (13). Recently, machine learning (ML) has demonstrated considerable advantages in the early detection and effective management of diseases (14–18). On one hand, ML can efficiently process vast amounts of data at relatively low costs; on the other hand, it can analyze these datasets to generate hypotheses and uncover latent patterns. Specifically, ML techniques have the capacity to extract common features from diverse data sources, such as text, images, behavioral data, and physiological indicators, thereby unveiling the intricate relationships between DKD and various contributing factors (19).

DKD is clinically diagnosed based on the albuminuria and the estimation of glomerular filtration rate (eGFR). However, DKD often has an insidious onset, with many patients being diagnosed only after irreversible renal damage has already occurred (4, 20). Recent studies have demonstrated that DKD is associated with a variety of metabolites, including those found in plasma, serum, urine, and mitochondria (21). Additionally, genetic variants linked to diabetes are frequently correlated with an increased risk of developing DKD (22). Retinal microvascular changes, such as retinopathy, vascular stenosis, dilation, and tortuosity, have also been shown to be associated with DKD (23). The potential of ML lies in its ability to integrate and analyze multidimensional data from diverse domains, including clinical, genetic, proteomic, metabolomic, and imaging information (24, 25). This capability holds significant promise for enhancing the accuracy of DKD risk prediction. Previous studies have demonstrated the feasibility of employing ML techniques to utilize various date types for identifying and predicting the risk of DKD in T2DM patients (26, 27).

In summary, ML algorithms hold potential value in predicting DKD risk. However, considering the diversity of ML algorithms, the initial differences in dataset characteristics, and the variations in sample sizes, the heterogeneity among studies cannot be overlooked. Moreover, although ML has garnered significant attention within the medical field, its robustness in clinical practice remains uncertain, and its widespread adoption and application are somewhat constrained. Therefore, in this systematic review and meta-analysis, we aimed to comprehensively integrate and evaluate the performance and clinical applicability of published ML-based models for predicting the DKD risk in T2DM patients. And hope to provide more reliable reference for clinical practice.

## 2 Materials and methods

### 2.1 Study design

This systematic review and meta-analysis were conducted in accordance with the guidelines recommended by the Cochrane Collaboration and the Preferred Reporting Items for Systematic Reviews and Meta-Analyses of Diagnostic Test Accuracy Studies (PRISMA-DTA) statement (28). The Checklist for Critical Appraisal and Data Extraction for Systematic Reviews of Prediction Modelling Studies (CHARMS) was employed to define the objectives of this systematic review and meta-analysis (29). The protocol for this systematic review and meta-analysis has been registered *a priori* at the International Platform of Registered Systematic Review and Meta-analysis Protocols (INPLASY) (30). (Registration: INPLASY202490038, https://doi.org/10.37766/inplasy2024.9.0038).

### 2.2 Date sources and search strategy

We aimed to compile predictive models for the DKD risk in T2DM patients based on ML algorithms, with the goal of evaluating their performance. With the assistance of information specialists, we conducted a comprehensive search across the following databases: PubMed, Embase, Cochrane Library, and Web of Science Core Collection. We included all relevant English-language publications up to April 18, 2024. Both controlled vocabulary terms (MeSH terms in Embase and PubMed) and free-text terms were employed using Boolean operators. The detailed search strategy is outlined in **Supplementary Table 1**.

### 2.3 Inclusion criteria

We included studies that evaluated the predictive performance of ML algorithms in assessing the DKD risk in T2DM patients, focusing on those that met the “PICOS” inclusion criteria.

#### 2.3.1 Participants

The participants were diagnosed with T2DM (31), with no eligibility restrictions based on gender, age, ethnicity, or geographical location. Studies focusing on other types of diabetes were excluded from this review.

#### 2.3.2 Intervention

Studies were included if they explicitly specified the application of clinical prediction models based on ML algorithms in T2DM patients. This included all relevant synonyms and methodologies related to ML, such as “supervised machine learning”, “unsupervised machine learning”, “deep learning”, “neural networks” and “support vector machines”. Consequently, studies that did not employ ML algorithms or those where ML was applied in nonclinical settings were excluded.

#### 2.3.3 Comparators

We included studies that compared ML algorithms with other ML algorithms, traditional statistical analyses, clinical scoring tools, and manual diagnoses with or without clinical scoring tools. Studies that solely used traditional statistical prediction tools or relied exclusively on unaided clinical performance were excluded.

#### 2.3.4 Outcomes

The primary outcome indicator is the risk of developing DKD in T2DM patients (1). Included studies must report model performance metrics, specifically area under the receiver operating characteristic curve (AUC).

#### 2.3.5 Study

Cohort studies, case-cohort studies, case-control studies and nested case-control studies.

### 2.4 Exclusion criteria

We excluded studies in the form of review articles, meta-analysis, case reports, conference abstracts, guidelines, editorials, commentaries, expert opinions, letters, and animal studies. Additionally, studies employing simple algorithms instead of ML were excluded. We also excluded studies that merely analyzed influencing factors without constructing a ML risk mode. Furthermore, studies that used ML exclusively for image recognition without developing a predictive model were excluded. In case where multiple studies used the same or overlapping patient datasets, only the most recent study was included.

### 2.5 Study selection

Firstly, the retrieved studies were imported into EndNote software for reference management where duplicate references were identified and removed. Secondly, two independent reviewers (Y.H. Li and N. Jin) performed a preliminary screening of the titles and abstracts of the included studies. This screening focused on selecting only those studies pertinent to the use of ML algorithms for risk prediction of DKD in T2DM patients. Note that two reviewers are required to independently evaluate the full research report and apply predefined inclusion and exclusion criteria to determine eligibility. Additionally, we manually searched the references of the included studies to identify any other potentially eligible studies. In cases of disagreement regarding the inclusion of a study during the screening process, consensus was achieved through discussion or by consulting a third independent reviewers (Q.Z. Zhan) for arbitration.

### 2.6 Date extraction

We employed the CHARMS checklist and the Transparent Reporting of a multivariable prediction model for Individual Prognosis or Diagnosis (TRIPOD) guidelines to develop standardized forms for data extraction (29, 32). Two independent reviewers (Y.H. Li and N. Jin) extracted data from the studies that were ultimately included. Notably, during the extraction process, if a single study reported multiple performance outcomes for the same ML model, we selected the best result. Additionally, if a study included two or more models, we extracted performance metrics for each model. Specifically, the following information was extracted from each study: first author, year of publication, country, data source, study design, research objectives, sample size, participant characteristics, age, gender distribution, type of prediction, type of ML model, category of predictive factors, model development and validation processes, and model performance metrics. The primary performance metrics of the included models typically included the area under the AUC, C-statistic, sensitivity, specificity, accuracy, F1 score, positive predictive value (PPV), and negative predictive value (NPV). In our study, AUC and the associated 95% confidence intervals (CI) were primarily used as the key metrics for evaluating model performance. If necessary, we attempted to contact the study authors for additional detailed information.

### 2.7 Risk of bias assessment

We assessed the risk of bias in the prediction models using the Prediction Model Risk of Bias Assessment Tool (PROBAST) (33), which is specifically designed for studies involving multivariable prediction models for individual prognosis or diagnosis. This tool assesses four domains: participants, predictors, outcomes, and statistical analysis. Each domain includes 2 to 9 key questions, with responses categorized as “Yes”/”Probably Yes”, “No”/”Probably No”, or “Unclear”. If at least one question within a domain is rated as “No” or “Probably No” the domain is considered high risk. Conversely, if all questions are rated as “Yes” or “Probably Yes” the domain is deemed low risk. When all domains are assessed as low risk, the overall risk of bias is categorized as low. If at least one domain is considered high risk, the overall risk of bias is rated as high. If the assessment is uncertain, the overall risk is marked as unclear. Similarly, this section was evaluated by two independent reviewers (Y.H. Li and N. Jin), who cross-checked their results. In cases of disagreement, a third reviewer (Q.Z. Zhan) was consulted to make the final decision.

### 2.8 Statistical analysis

We conducted a meta-analysis, pooling the AUC values and their 95% CIs from individual studies, and performed stratified analyses based on study design, model type, and other relevant factors. If the AUC did not report a 95% CI or standard error (SE), we estimated the SE and 95% CI using the Hanley and McNeil formula (34–36). Given the high heterogeneity among the included studies due to variations in study design, ML models, predictive factors, and parameters, we used the Der Simonian and Laird random-effects model to pool the AUCs in the meta-analysis (37). Additionally, we calculated the 95% prediction interval (PI) to characterize the degree of heterogeneity among studies and to assess the potential range of the predictive model’s performance in future studies. The results were presented in the form of a forest plot. Moreover, we assessed the degree of heterogeneity between studies using the Cochrane Q test and *I²* statistic to determine the suitability of a fixed-effects model (*P* < 0.10 or *I*² > 25%) (38). It is important to note that when the 95% CI or PI of the pooled AUC includes 0.5, we consider there to be insufficient evidence to demonstrate statistically significant discriminatory ability of the prediction model for DKD occurrence in the populations included in the meta-analysis. AUC values were classified as follows: <0.60 as inadequate, 0.60–0.70 as moderate, 0.70–0.80 as acceptable, and >0.80 as excellent. All statistical analyses were conducted using STATA version 18. Statistical significance was defined as a *P*-value less than 0.05, with a threshold of 0.10 for heterogeneity testing.

## 3 Results

### 3.1 Study selection

In this study, two independent reviewers (Y.H. Li and N. Jin) conducted a comprehensive screening and integration of the data. The inter-rater reliability was assessed using the Kappa coefficient, which indicated substantial agreement between the two reviewers (kappa = 0.82).

**Figure 1:**
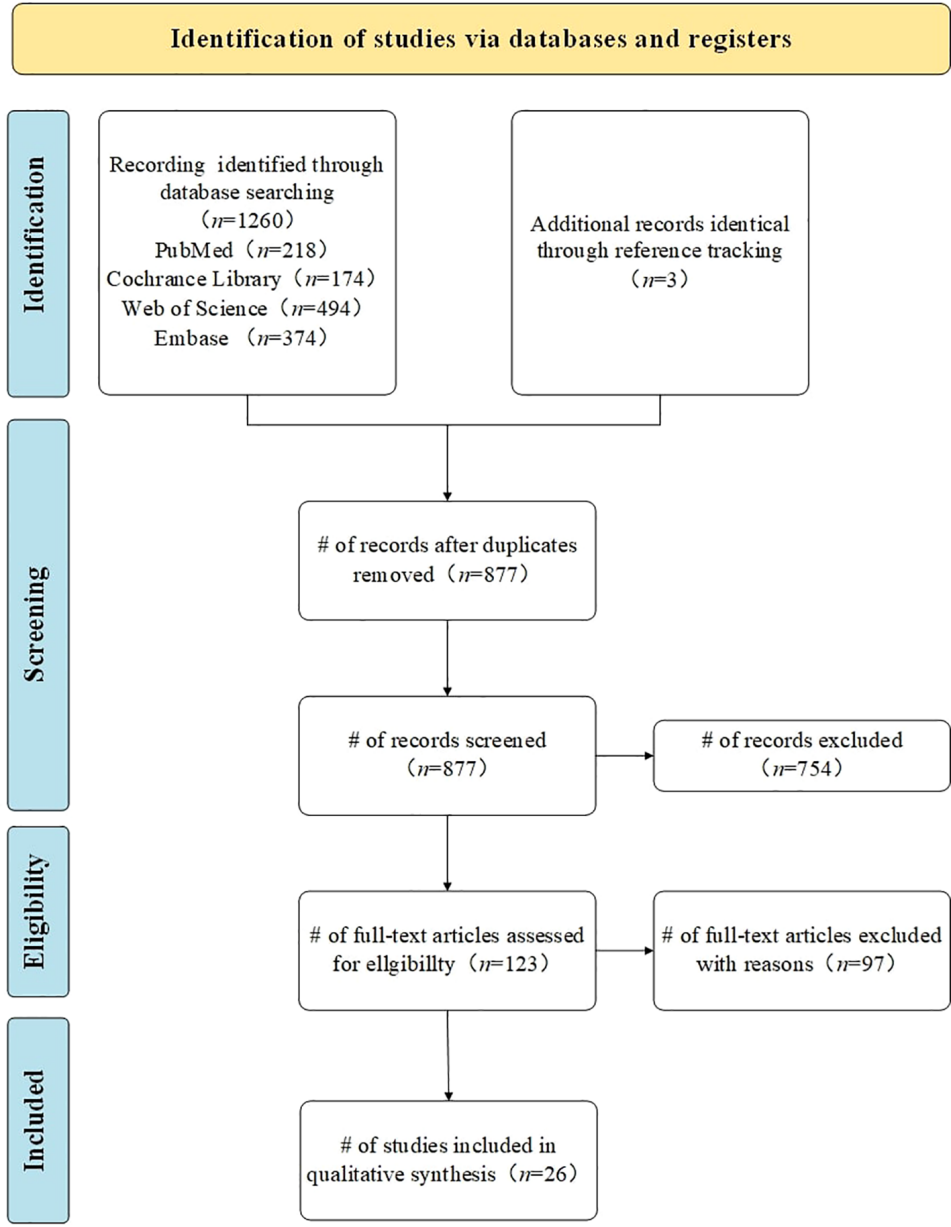
Study search and selection flowchart. Flowchart according to the Preferred Reporting Items for Systematic Reviews and Meta-Analyses (PRISMA 2020). *n*= number of studies/records/reports.

A total of 1260 studies were identified through a systematic search of the predetermined electronic databases, based on the established search strategy. Specifically, we retrieved 218 records from PubMed, 174 records from the Cochrane Library, 494 records from Web of Science, and 374 records from Embase. Additionally, we identified 9 studies through reference list reviews. Subsequently, after removing duplicates, 877 studies remained. We screened these studies by reviewing titles and abstracts, excluding 754 studies that either reported irrelevant topics or lacked predefined outcomes. The remaining 123 studies were further assessed through full-text review. Finally, 26 studies meeting the inclusion and exclusion criteria were included for meta-analysis (26, 27, 39–62). A flowchart of the study search and selection process is detailed in **Figure 1**, and search information from each electronic database is provided in **Supplementary Table 1**.

### 3.2 Study characteristics

Among the 26 studies included, 14 were from China (39–41, 43, 48, 49, 51–53, 55, 59, 60), 4 from the United States (42, 50, 56, 58), 3 from Singapore (26, 44, 45), 3 from Iran (46, 47, 54), and 1 each from Italy (27) and Bangladesh (57). Only one study was published in 2013 (61), while the rest were published in the past five years. All studies were predominantly retrospective, comprising 14 cohort studies (26, 41, 42, 46, 48–52, 55, 56, 58, 61, 62), 9 cross-sectional studies (39, 40, 45, 47, 53, 54, 57, 59, 60), and 3 case-control studies (27, 43, 44). The basic characteristics of each study are detailed in **Table 1**.

**Table 1:** Characteristics of studies included in the systematic review and meta-analysis.

Study	Country	Study Type	model	Sample Size	Male/ Female	Age	Predictors	Machine Learning	Performance Evaluation	Validation
Zhao et al. (2023) (39)	China	Cross	DI	Total: 678 Training:202 Internal validation:87 External validation:389	467/211	54.1 ± 11.8	MH+LE	LR	AUC, Accuracy, Sensitivity, Jordan index	INV+EXV
Liu et al. (2020) (40)	China	Cross	DI	Total: 1485(10-fold cross) Training: Internal validation:	N/A	N/A	DC+LE+CI	BN, BN-wopi, NB, RF, DT	AUC, Sensitivity, Specificity	INV
Hui et al. (2023) (41)	China	Cohort	DI	Training:241	100/141	52.57 ± 10.65	MH+LE	LASSO	AUC, Precision score, Recall score, Accuracy	INV
Momenzadeh et al. (2022) (42)	USA	Cohort	PR	Total: 10468 Training:8388 Internal validation: 2097	5067/5401	57.7(48.3-68.3)	DC+LE+SH	SVC, GBDT, ET, AdaBoost, RF, LR	AUC	INV
Cai et al. (2024) (43)	China	Case	DI	Total: 210 Training:147 Internal validation:63	87/123	57.42 ± 8.15	MH+LE+CI	DT, RF	AUC, sensitivity, specificity, accuracy, recall, precision	INV
Betzler et al. (2023) (44)	Singapore	Case	DI	Training:13284(5-fold cross) Internal validation (5-fold cross); External validation: SEED:1969; SMART2D:712	6758/6526 1047/922 380/332	64.1 ± 10.8 64.0 ± 9.2 57.4 ± 10.7	DC+MH+LE+RP	DL	AUC, sensitivity, specificity, PPV, NPV	INV+EXV
He et al. (2024) (45)	Singapore	Cross	DI	Total: 2772(5-fold cross) Training:2219 Internal validation:553 External validation:5843	1411/1361 3753/2090	61.7(53.5-69.4) 61.0(55.0-65.0)	DC+MH+DD+PD+SH+LE	LASSO, GBDT, LR	AUC, sensitivity, specificity	INV+EXV
Hosseini Sarkhosh et al. (2023) (46)	Iran	Cohort	PR	Total: 1907 Training:1526 Internal validation:381 External validation:1543	854/1053 683/860	58.4 ± 9.4 58.63 ± 9.24	DD+MH+CI	LR	AUC, accuracy, precision, f1score	INV+EXV
Afrash et al. (2022) (47)	Iran	Cross	DI	Total: 327 (5-fold cross) Training: Internal validation:	176/151	54.7 ± 3.8 68.23 ± 5.6	DC+DD+LE+PE	DT	sensitivity, spec-ificity, accuracy	INV
Yang et al. (2024) (48)	China	Cohort	DI	Total: 162 Training: 114 Internal validation: 49	100/63	58 ± 12	RS	DL	ROC, accuracy, specificity, sensitivity, PPV, NPV	INV
Yin et al. (2024) (49)	China	Cohort	DI	Total: 562 (10-fold cross) Training: 9 subsets Internal validation: 1 subset	N/A	N/A	MH+DD+PD+PE+LE	XGB, RF, DT, LR	AUC-ROC, Accuracy	INV
Song et al. (2020) (50)	USA	Cohort	PR	Total: 14039 Training: 11231 Internal validation: 2808	6786/7253	58 ± 18	DC+LE+PE+RS+SH	GBM	AUROC, AUPRC	INV
Fan et al. (2021) (51)	China	Cohort	DI	Total: 165 Training: 132 Internal validation:33	97/68	N/A	DC+MH+FH+DD+PD	BN, CHAID, D, XF	AUC, NPV, PPV, accuracy.	INV
Zhang et al. (2022) (52)	China	Cohort	DI	Total: 929(5-fold cross) Training: 743 Internal validation:186 External validation:329	608/321 234/94	51.34 ± 10.02 51.83 ± 9.66	DD+CI+LE+FH	RF, SVM	AUC, Accuracy, Sensitivity, Specificity, PPV, NPV, Balanced accuracy	INV+EXV
Liu et al. (2023) (53)	China	Cross	DI	Total: 3624 Training: 2899 Internal validation:725	2034/1590	59 ± 9.0	DC+SH+PE+LE	CatBoost, GBM, XGBoost, ET, GBDT, RF, LDA, LR, QDA, AdaBoost, NB, KNN, DT, SVM, RC	AUC, Accuracy	INV
Hosseini Sarkhosh et al. (2022) (54)	Iran	Cross	DI	Total: 6235 Training: 4988 Internal validation:1247	2754/3481	57.6 ± 11.9	DC+DD+LE+PE+CI	DT, SVM, LR, RF, XGBoost	AUC	INV
Dong et al. (2022) (55)	China	Cohort	PR	Total: 2809 Training: 652 Internal validation:164	585/231	56(48.3-65.0)	DC+LE+PE	GBM, XGBoost, AdaBoost, ANN, DT, SVM, LR	AUC, sensitivity, specificity, accuracy, F1	INV
Nicolucci et al. (2022) (27)	Italy	Case	PR	Total: 147664(Ten-fold Cross) Training: nine folds Internal validation: one fold	N/A	N/A	N/A	XGBoost	AUC, accuracy, sensitivity, specificity	INV+EXV
Sabanayagam et al. (2023) (26)	Singapore	Cohort	PR	Total: 1365 Training: 652 Internal validation:164	698/667	58.74 ± 8.95	DC+MH+PD+CI+LE+PE	LASSO, EN, GBDT	AUC	INV
Allen et al. (2022) (56)	USA	Cohort	PR	Total: 87963 Training: 62994 Internal validation:17323 Hold-out testing sets: 7656 External validation: 23073	N/A	N/A	DC+MH+LE+SH+FH	RF, XGBoost	AUC, Sensitivity Specificity Positive and negative likelihood ratios, DOR	INV+EXV
Maniruzzaman et al. (2021) (57)	Bangladesh	Cross	DI	Training:133	55/78	54.3 ± 12.5	DC+DD+LE+PE	LDA, SVM-RBF, LR, KNN, NB	AUC, sensitivity, specificity, accuracy, F1	INV
Song et al. (2019) (58)	USA	Cohort	DI	Total: 15645 Training: 8089 Internal validation: 3461 External validation: 4086	7726/7919	59.0 ± 14.0	DC+DD+LE+PE+CI	GBM	AUC, sensitivity, specificity, accuracy, F1	INV+EXV
Wang et al. (2021) (59)	China	Cross	DI	Training:2163	1227/949	57.27 ± 11.93	DC+MH+DD+SH+PE	LR	AUC	INV
Shi et al. (2023) (60)	China	Cross	DI	Total: 512 Training: 360 Internal validation:155	329/186	54.1 ± 0.64	DC+MH+DD+LE+PE	RF, SVM, GBDT, Adaboost	AUC	INV
Leung et al. (2013) (61)	China	Cohort	PR	Total: 673 Training: 360 Internal validation:155	277/396	57 ± 8.5	DC+DD+LE+PE+GA	SVM, Cforest	AUC, sensitivity, specificity, accuracy, F1	INV
Su et al. (2023) (62)	China	Cohort	DI	Total: 219 Training: 175 Internal validation:44	140/79	56.15 ± 10.98	DC+LE	KNN, SVM, LR	sensitivity, specificity, accuracy, F1, MCC, AUC	INV

Cross, cross-sectional study; Cohort, Cohort Study; Case, Case-Control Study; DI, diagnostic model; PR, prognostic model; MH, medical history; LE, laboratory examinations; DC, demographic characteristics; CI, complications information; SH, social-lifestyle history; RP, Retinal Photographs; DD, Diabetes duration; PD, previous drug used; PE, Physical Examination; RS, Radiomic Signatures; FH, family history; GA, genetic attributes; CM, circulating metabolites; RH, renal histopathological results; LR, logistic regression; RF, random forest; DT, decision tree; SVM, support vector machine; GBDT, gradient boosted decision trees; BN, Bayesian networks; NB, Naive Bayes; AdaBoost, Adaptive Boosting; LASSO, Least Absolute Shrinkage and Selection Operator; MICE, Multivariate Imputation by Chained Equations; RFECV, Recursive Feature Elimination with Cross-Validation; GA, Genetic Algorithm; PCA, Principal Component Analysis; XGBoost, Extreme Gradient Boosting; KNN, K-Nearest Neighbors; AUC, area under the receiver operating characteristic curve; INV, Internal validation; EXV, External validation.

Most of the data were obtained from electronic health record systems (EHRs) in hospitals, with a smaller proportion sourced from specialized disease research public databases, such as the Singapore Eye Disease Epidemiology Study Database and the Diabetic Retinopathy Comprehensive Project (26, 44, 45, 54). The included variables typically encompass demographic information (e.g., gender, age), disease characteristics (e.g., medical history, disease duration, complications), lifestyle factors (e.g., smoking, alcohol consumption), physical examination measures (e.g., height, weight), and clinical laboratory test results (e.g., blood and urine analysis). Additionally, some studies incorporated circulating metabolites (26, 45) and genetic parameters (26, 56) as variables. Furthermore, some studies used unstructured data, such as renal pathology images (39), retinal color fundus photographs (26, 44, 60), and renal ultrasound images (48).

Regarding the types of predictive models, 18 studies focused on diagnostic models (39–41, 43–45, 47–49, 51–54, 57–60, 62), while the remaining 8 were prognostic models (26, 42, 46, 50, 55, 56, 61). Among the prognostic models, the study by Leung et al. (61) had the longest median follow-up period of 7.8 years, whereas the study by Song et al. (50) had a follow-up period of only 1 year.

Twenty-five studies conducted internal validation, using either holdout validation sets or K-fold cross-validation. The total sample size across all internal validation was 319,190 participants. However, there was substantial variation in sample sizes among the studies, with Nicolucci et al. (27) having the largest sample size of 147,664, and Maniruzzaman et al. (57) having the smallest with 133. External validation was performed in 8 studies (27, 39, 44–46, 52, 56, 58). 4 studies employed external validation using datasets independent of the training set (27, 39, 44, 45). 3 studies used a temporal split method, dividing data chronologically into training and external validation sets, with earlier data used for training and later data for external validation (46, 52, 58). For instance, Hosseini Sarkhosh et al. (46) used data from 2012-2016 as the training set and data from 2017-2021 as the external validation set. Additionally, while the internal and external validation sets in the study by Allen et al. (56) were derived from the same large dataset, the external validation set consisted solely of clinical data, which was independent of the training set. 7 of those external validation had a combined external validation sample size of 37,944. One study conducted external validation across 5 different centers (27), with reported cohort sizes ranging from 3,912 to 200,007, though specific sample sizes for the other centers were not provided.

### 3.3 ML models and performance evaluation

There were 26 ML models involved across 26 studies. The most commonly applied ML algorithms included logistic regression (LR, *n* = 11), random forest (RF, *n* = 9), decision tree (DT, *n* = 9), support vector machine (SVM, *n* = 6), gradient boosted decision trees (GBDT, *n* = 5), Bayesian networks (BN, *n* = 3), Naive Bayes (NB, *n* = 3), Adaptive Boosting (AdaBoost, *n* = 3), and Least Absolute Shrinkage and Selection Operator (LASSO, *n* = 3). All studies used classical methods to assess the discriminative ability of ML models. We used AUC and associated 95% CI as the primary ability metrics. Additionally, other ability evaluation metrics included Accuracy, Specificity, Sensitivity, Jordan index, Precision score, Recall score, PPV, and NPV, and F1 score.

### 3.4 Handling of missing data

Most studies addressed missing data using various imputation methods. Among them, six studies used mean or median imputation (45, 46, 52, 54, 56, 61). Momenzadeh et al. (42) applied the MissForest imputation method, while Liu et al. (40) employed the Multivariate Imputation by Chained Equations (MICE) algorithm. Additionally, 2 studies used ML algorithms for imputation (55, 60). However, 3 studies only mentioned the use of imputation without specifying the exact methods employed (27, 50, 53). Three studies excluded missing data directly (39, 44, 48), while 6 studies selectively excluded missing data based on a certain proportion (26, 41, 47, 49, 51, 58). There were also 3 studies that did not mention their method to handling missing data (57, 59, 62). Furthermore, one prospective study had no missing data, and thus, did not involve any imputation methods (43).

### 3.5 Methods of selecting predictors

Of the studies reviewed, two did not mention the method used for predictor selection (27, 44), one used all available features as predictors (42), and another determined predictors based on existing literature and expert consensus (56). The remaining 22 studies detailed their methods for predictors selection. The most frequently employed method was Recursive Feature Elimination with Cross-Validation (RFECV), followed by the LASSO. Additionally, some studies used multivariate LR, the Markov Blanket, the Genetic Algorithm (GA) feature selection method, and various ML algorithms, including RF, GBDT, Gradient Boosting Machine (GBM), SVM, DT, Extreme Gradient Boosting (XGBoost), and Principal Component Analysis (PCA) et al. for selecting predictors.

### 3.6 Quality of evidence and risk of bias

All included studies were assessed for risk of bias using the PROBAST tool. Overall, with the exception of two studies that were assessed as low risk and one study that was assessed as unclear risk, the remaining studies were assessed as having a high risk of bias. In domain 4 (statistical analysis), a significant proportion of studies had a high risk of bias. In particular, 14 studies failed to properly assess the discrimination and calibration of the prediction models, which is a common source of bias. Furthermore, 53.8% of the studies did not assess whether the coefficients and intercepts of the final predictors in the development phase were consistent with the results reported in the multivariable analysis, another common bias. Although the vast majority of studies (96.1%) conducted internal validation, a portion of these studies (34.6%) relied solely on random data splitting as internal validation, which cannot be considered a proper application of internal validation methods. This leading to an increased risk of bias. Eleven studies (42.3%) did not provide adequate explanations for handling complex data. Eight studies (30.8%) failed to appropriately address missing data in their included subjects. Additionally, 8 studies (30.8%) had unreasonable sample size designs, with 4 studies having an event-per-variable (EPV) ratio of less than 10 during the development of predictive models, and another 4 studies having a validation cohort sample size of fewer than 100 cases. These results indicate that potential bias may be present in the majority of the included studies, which should be considered carefully when interpreting the results. A summary table of the results from the PROBAST assessment can be seen in **Supplementary Table 2**.

### 3.7 Statistical analysis

**Figure 2:**
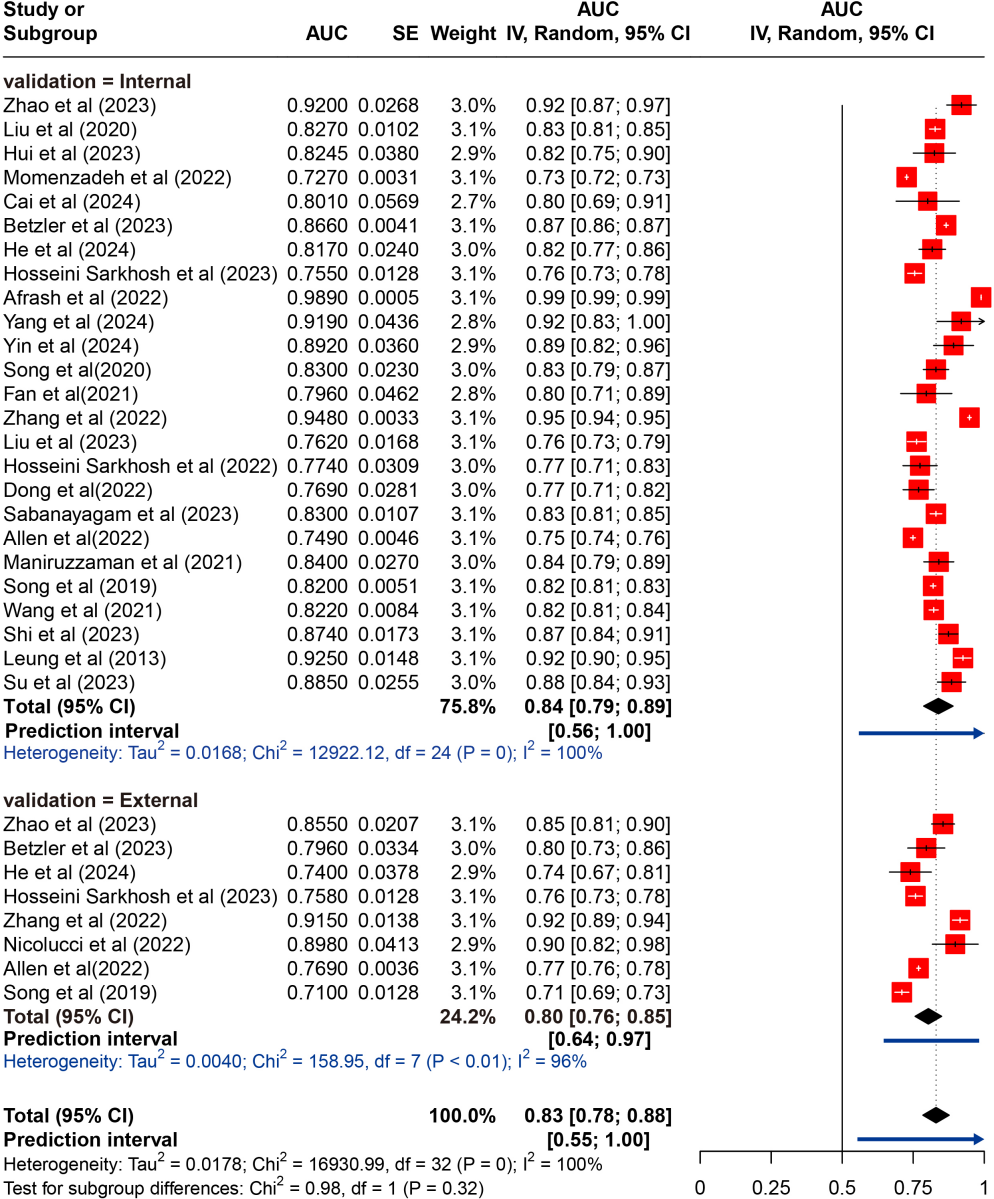
Random effects forest plot of area under the curve (AUC) values with 95% confidence intervals (CI) in internal and external validation. AUC, area under the receiver operator characteristic curve. The weight % represents the contribution of each study to the overall analysis. The diamond shapes indicate pooled AUC estimates for each subgroup (internal, external) and the overall analysis. Horizontal lines represent the confidence intervals for individual studies, and the dashed vertical line indicates the pooled AUC estimate. p values from Cochran’s Q test.

Twenty-five studies underwent internal validation, while only 8 studies performed external validation (27, 39, 44–46, 52, 56, 58). A total of 94 ML models were developed, with 81 models in the internal validation set and 13 models in the external validation set. The meta-analysis of the AUC included data from 25 studies for internal validation sets and 8 studies for external validation sets. Since most studies employed multiple ML models, we first pooled their AUC, as shown in **Supplementary Table 3**. A meta-analysis was then subsequently performed using the pooled AUC from each study. The pooled AUC in the internal set was 0.839 (95% CI 0.787-0.890; *I*²= 99.8%; *P* = 0.000 for heterogeneity), with a 95% PI ranging from 0.56 to 1.00. In contrast, the pooled AUC in the external validation set was 0.830 (95% CI 0.784-0.877; *I*²= 95.6%; *P* = 0.000 for heterogeneity), with a narrower 95% PI of 0.64-0.97 that excluded 1, indicating more stable performance and better generalizability across external datasets, despite significant heterogeneity. Moreover, these results indicated a marginally higher pooled AUC in the internal validation set compared to the external validation set. The results of the meta-analysis were presented in **Figure 2**.

To identify potential sources of heterogeneity, we conducted subgroup analyses based on study type and prediction model type using the internal validation datasets. Individually, the pooled AUC for cross-sectional studies was 0.848 (95% CI 0.770-0.926; *I*²= 99.2%; *P* = 0.000 for heterogeneity), for cohort studies it was 0.833 (95% CI 0.775-0.891; *I*²= 99.5%; *P* = 0.000 for heterogeneity). Notably, case-control demonstrated a pooled AUC of 0.858 (95% CI 0.817-0.900) with significantly reduced heterogeneity (*I*² = 23.0%; *P* = 0.254). When grouped by prediction model type, pooled AUC was 0.797 (95% CI 0.757-0.836; *I*²= 97.7%; *P* = 0.000 for heterogeneity) for prognostic models and 0.856 (95% CI 0.815-0.896; *I*²= 99.5%; *P* = 0.000 for heterogeneity) for diagnostic models. The results of the meta-analysis were presented in **Supplementary Figures 1**, **2**.

All used algorithms according to the history of ML into traditional regression ML models (*n* = 14), ML models (*n* = 21), and deep learning (DL) models (*n* = 4). Traditional regression ML models refer to those that use regression techniques of ML, including LR and LASSO. ML models are more complex than traditional regression ML models (such as RF, DT, SVM, et al). Additionally, we defined DL models as neural networks with two or more hidden layers, such as CNN and ANN. We then pooled their AUC. The results indicated that the pooled AUC for traditional regression ML models was 0.797 (95% CI 0.777-0.816) with significant heterogeneity (*I*²= 96.7%, *P* = 0.000), for ML models was 0.811 (95% CI 0.785-0.836; *I*²= 99.9%, *P* = 0.000 for heterogeneity). In contrast, the pooled AUC for DL models was 0.863 (95% CI 0.825-0.900; *I*²= 81.1%, *P* = 0.000 for heterogeneity), indicating a outperformed both ML and traditional regression ML models. The results of the meta-analysis were presented in **Supplementary Figure 3**.

**Figure 3:**
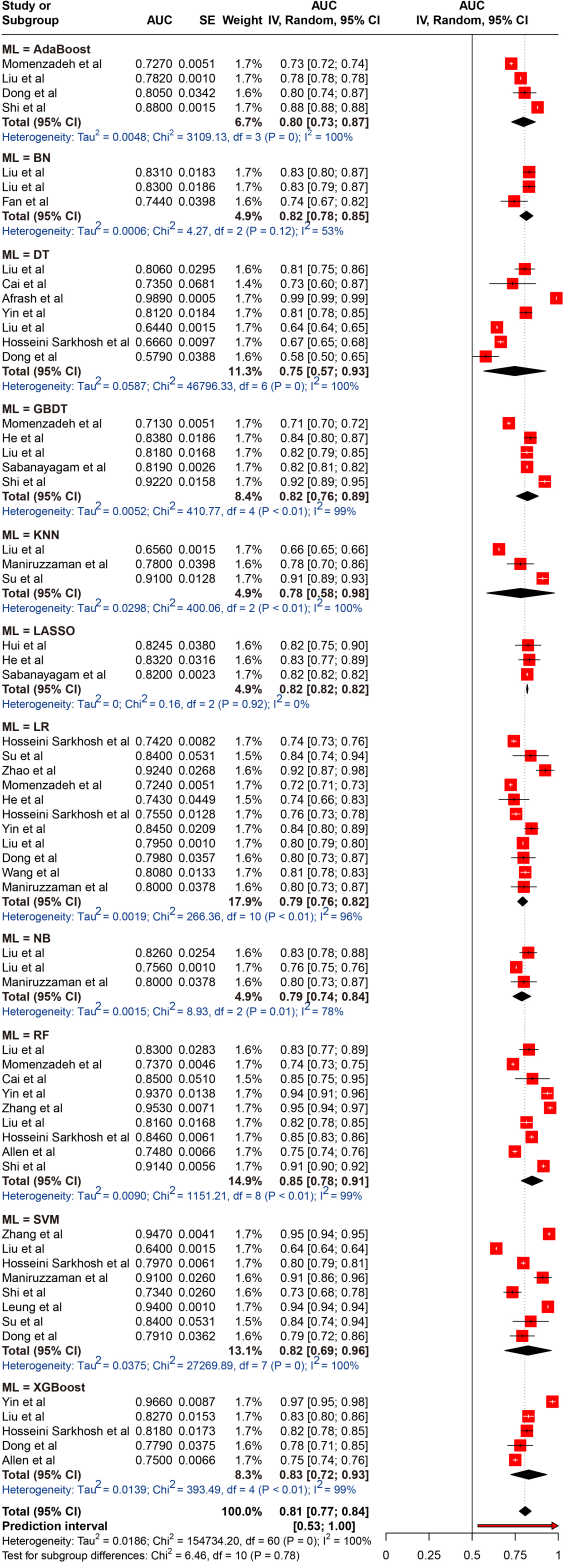
Random effects forest plot of area under the curve (AUC) values with 95% confidence intervals (CI) in machine learning (ML) type. AUC, area under the curve; AdaBoost, Adaptive Boosting; BN, Bayesian networks; DT, decision tree; GBDT, gradient boosted decision trees; LASSO, Least Absolute Shrinkage and Selection Operator; LR, logistic regression; NB, Naive Bayes; RF, random forest; SVM, support vector machine; XGBoost, Extreme Gradient Boosting. The weight % represents the contribution of each study to the overall analysis. The diamond shapes indicate pooled AUC estimates for each subgroup (internal, external) and the overall analysis. Horizontal lines represent the confidence intervals for individual studies, and the dashed vertical line indicates the pooled AUC estimate. p values from Cochran’s Q test.

A total of 26 different ML models were analyzed. To identify which models performed best, we pooled and compared the AUC of those models that were used three or more times. The results indicated that the pooled AUCs were as follows: AdaBoost, 0.798 (95% CI 0.729-0.868); BN, 0.815 (95% CI 0.777-0.854); DT, 0.748 (95% CI 0.567-0.929); GBDT, 0.821 (95% CI 0.757-0.885); LASSO, 0.820 (95% CI 0.816-0.825); LR, 0.793 (95% CI 0.764-0.823); NB, 0.788 (95% CI 0.737-0.840); RF, 0.848 (95% CI 0.785-0.911); SVM, 0.825 (95% CI 0.690-0.960); and XGBoost, 0.829 (95% CI 0.724-0.934). Among the models, the RF exhibited the best performance, while the DT model had the lowest performance; however, these differences were not statistically significant. Despite the high degree of heterogeneity, the 95% PIs for ML models demonstrated statistical significance (0.53–1.00). The results of the meta-analysis were presented in **Figure 3**.

### 3.8 Sensitivity analysis

A total of 25 studies were included, and excluding any single study did not result in a statistically significant difference in the pooled results from the remaining studies (*n* = 24). The results remained consistent with the original pooled AUC (0.84, 95% CI 0.79-0.89), indicating the stability of the findings. The results of the sensitivity analysis were presented in **Supplementary Figure 4**.

### 3.9 Predictors screened

The final predictors selected for developing the ML model can be categorized into 14 types: demographic characteristics, medical history, T2DM duration, previous drug use history, complication information, social lifestyle history, family history, physical examination, laboratory examinations, renal histopathological results, radiomic signatures, circulating metabolites, genetic attributes, and retinal photographs. Based on these studies, we can identify several risk factors for the development of DKD in patients with T2DM. These include age, gender, race; history of hypertension, cardiovascular disease, cerebrovascular disease; T2DM duration; smoking, alcohol; family history of diabetes; use of antidiabetic (e.g., insulin, glinides, TZDs) and antihypertensive medications; complications such as diabetic retinopathy and diabetic peripheral vascular disease; physical examination parameters like height, weight, BMI, pulse pressure, systolic blood pressure (SBP), diastolic blood pressure (DBP), and waist-to-hip ratio; Additionally, laboratory examinations result such as 2-hour Postprandial glucose (2hPPG), glucose, insulin, fasting blood glucose (FBG), hemoglobin A1c, low-density lipoprotein (LDL), high-density lipoprotein(HDL), triglycerides, total cholesterol; circulating metabolites like tyrosine, lactate, intermediate-density lipoprotein cholesteryl ester % (IDL-CE%); and citrate, genetic attributes including uteroglobin gene G38A mutation (UGB G38A), LIPC514C > T, and apolipoprotein B threonine to isoleucine substitution at position 71 (APOBThr71Ile); as well as renal histopathological results; retinal photographs, and renal radiomic signatures. We have integrated these predictors, as shown in **Table 2**.

**Table 2:** Predictors included in the machine learning model.

Feature	Final Predictors
Demographic characteristics	Age, Gender, Race
Medical history	Hypertension, Cardiovascular disease, Cerebrovascular disease
Duration	T2DM duration
Social lifestyle history	Smoking, Alcohol
Family history	Family history of diabetes
Histopathological	Renal histopathological results
Radiomic signatures	Renal radiomic signatures
Photographs	Retinal Photographs
Circulating metabolites	Tyrosine, Lactate, DL-CE%, Citrate, Glycoprotein acetyls, LLDL-PL%, Alanine.
Genetic attributes	(Urinary Glucose Blood Glycine 38 to Alanine Mutation) UGB G38A, Lipase C Gene Mutation (514C > T) (LIPC514C > T), Apolipoprotein B Threonine 71 to Isoleucine Mutation (APOBThr71Ile), Apolipoprotein C3 Gene Mutation (3206T > G) (APOC33206T > G) and Apolipoprotein C3 Gene Mutation (1100C > T) (APOC31100C > T)
Previous drug use history	Antidiabetic medications: insulin use, TZDs, glinides, DPP-4 inhibitors, GLP-1 receptor agonists, SGLT-2 inhibitors; Antihypertensive medications
Complication information	Diabetic retinopathy Diabetic peripheral vascular disease Macrovascular complications of diabetes
Physical examination	Height, Weight, BMI, Pulse pressure, SBP, DBP, DAP, SAP
Laboratory examinations	2hPPG, GLU, INS, FBG, HbA1c; LDL, HDL, TG, TC, Apo-A, Apo-B, APB: APA; BUN, CREA, BUN/CREA, UA, eGFR, 24h-UTP, MAU, ACR, CYS-C, hematuria, significant proteinuria; ALB, GLB, ALP, γGGT, ALT, AST; HCT, WBC, PLT, PDW, MCHC, HGB, PCV, RDW-SD, RDW, MPV, Mon%, Bas, Eos, Neu, P-LCR, Lym; FT3, TSH, IGF-1, FIB, INR, Tyr, C2, C4DC, C5DC, C24, Na, K, PP, PLT, PT, TT, Bicarbonate, DHA, LLDL-CE%, IDLC%.

T2DM, Type 2 Diabetes Mellitus; DL-CE%, Double Layer Cholesterol Ester Percentage; LLDL-PL%, Large Low-Density Lipoprotein Phospholipid Percentage; UGB, Urinary Glucose Blood; G38A, Glycine 38 to Alanine Mutation; LIPC514C > T, Lipase C Gene Mutation (514C > T); APOBThr71Ile, Apolipoprotein B Threonine 71 to Isoleucine Mutation; APOC33206T > G, Apolipoprotein C3 Gene Mutation (3206T > G); APOC31100C > T, Apolipoprotein C3 Gene Mutation (1100C > T); TZDs, Thiazolidinediones; DPP-4, Dipeptidyl Peptidase-4; GLP-1, Glucagon-Like Peptide-1; SGLT-2, Sodium-Glucose Cotransporter-2; BMI, Body Mass Index; SBP, Systolic blood pressure; DBP, Diastolic blood pressure; DAP, Waist-to-hip ratio, Diastolic arterial pressure; SAP, Systolic arterial pressure; 2hPPG, 2-hour Postprandial Plasma Glucose; GLU, Glucose; INS, Insulin; FBG, Fasting Blood Glucose; HbA1c, Hemoglobin A1c; LDL, Low-Density Lipoprotein; HDL, High-Density Lipoprotein; TG, Triglycerides; TC, Total Cholesterol; Apo-A, Apolipoprotein A; Apo-B, Apolipoprotein B; APA, Apoptosis-Associated Protein; BUN, Blood Urea Nitrogen; CREA, Creatinine; BUN/CREA, Blood Urea Nitrogen to Creatinine Ratio; UA, Uric Acid; eGFR, Estimated Glomerular Filtration Rate; 24h-UTP, 24-hour Urinary Total Protein; MAU, Microalbuminuria; ACR, Albumin-to-Creatinine Ratio; CYS-C, Cystatin C; ALB, Albumin; GLB, Globulin; ALP, Alkaline Phosphatase; γGGT, Gamma-Glutamyl transferase; ALT, Alanine Aminotransferase; AST, Aspartate Aminotransferase; HCT, Hematocrit; WBC; White Blood Cell Count; PLT, Platelet Count; PDW, Platelet Distribution Width; MCHC, Mean Corpuscular Hemoglobin Concentration; HGB, Hemoglobin; PCV, Packed Cell Volume; RDW-SD, Red Cell Distribution Width-Standard Deviation; RDW, Red Cell Distribution Width; MPV, Mean Platelet Volume; Mon%, Monocyte Percentage; Bas, Basophil Count; Eos, Eosinophil Count; Neu, Neutrophil Count; P-LCR, Platelet Large Cell Ratio; Lym, Lymphocyte Count; FT3, Free Triiodothyronine; TSH, Thyroid-Stimulating Hormone; IGF-1, Insulin-Like Growth Factor-1; FIB, Fibrinogen; INR, International Normalized Ratio; Tyr, Tyrosine; C2, Carnitine C2 (Acetylcarnitine); C4DC, Butyrylcarnitine (C4 Dicarboxylate); C5DC, Glutarylcarnitine (C5 Dicarboxylate); C24 - Tetracosanoylcarnitine (C24); PP, Pancreatic Polypeptide; PT, Prothrombin Time; TT, Thrombin Time; Bicarbonate, Bicarbonate Ion; DHA, Docosahexaenoic Acid; LLDL-CE%, Large Low-Density Lipoprotein Cholesterol Ester Percentage; IDLC%, Intermediate-Density Lipoprotein Cholesterol Percentage.

## 4 Discussion

This meta-analysis systematically evaluated the application of ML-based risk prediction models for assessing the risk of DKD in patients with T2DM. We conducted a comprehensive analysis of 26 studies, exploring critical aspects such as study quality, the stability of internal and external validation, the performance of various ML models, and the types of predictive factors used.

Early prediction and identification of DKD are crucial for preserving renal function in patients with T2DM. This can lead to timely therapeutic interventions and lifestyle modifications, preventing disease progression to advanced stages and reducing dependence on dialysis and high healthcare expenditures (63). However, DKD often manifests insidiously. Our analysis indicated that ML demonstrates promising performance in predicting and identifying DKD in T2DM patients. The pooled AUC from internal validation sets was 0.839 (95% CI 0.787-0.890), and for external validation, it was 0.830 (95% CI 0.784-0.877). These findings suggest that our study could provide valuable insights for early clinical prediction of DKD.

It is important to note that the pooled AUC demonstrates considerable heterogeneity. This heterogeneity among subgroups may stem from variations in data sources and types. The studies included in this analysis draw upon diverse datasets, encompassing prospective clinical sample collections, publicly available datasets, and electronic health records. The data types also vary, including clinical laboratory tests, blood metabolomics, imaging, and genomics. All of which may contribute to the observed heterogeneity. Moreover, variations in the assessment tools for DKD, the demographic characteristics of the study populations, and the types of research designs may contribute to the heterogeneity across studies. In our analysis, the sample sizes of the included studies varied significantly, ranging from 133 to 147,664 participants. While sample size is an important factor, the proportion of positive cases is arguably more critical, as it determines the weight of each study. Consequently, a larger sample size may not directly influence the AUC value. However, studies with smaller sample sizes may experience a reduction in machine learning model performance, which could also serve as a potential source of heterogeneity.

Despite the significant potential demonstrated by extant studies in the application of machine learning for multidimensional data processing and risk prediction, the lack of external validation using completely independent datasets remains a major methodological issue in risk prediction model research (64, 65). Similarly, among the 26 studies included in our review, only 8 conducted external validation (27, 39, 44–46, 52, 56, 58), while the remaining studies did not employ external validation. External validation is imperative for evaluation of a model’s robustness and generalizability (66–68). It not only evaluates the model’s predictive ability but also tests its adaptability to new environments or different datasets, thereby revealing its effectiveness and limitations in real-world clinical practice (69). Although the negligible discrepancy (AUC values from internal and external validation sets differed by only 0.009), the absence of external validation hinders our understanding of the actual capability of the generalizability in our pooled models. This limitation restricts the applicability of our findings to diverse regions, environments, and healthcare systems. In addition, model overfitting or selectivity bias can result in good performance on the training set but poor performance on the external validation set (70). External validation can reveal whether the model has overfitted the training set, as well as selective bias during internal validation. In our study, it is also not yet clear whether there is overfitting or selective bias in the training set data due to the lack of external validation.

ML enhances model performance by synthesizing complex relationships among variables, surpassing traditional statistical analysis methods (67). Currently, ML algorithms are widely applied in the healthcare industry, demonstrating significant advantages in disease risk prediction. Another meta-analysis by Saputro et al. (8) focused solely on the performance of traditional Cox regression and LR models, without reporting on other ML techniques. Our study is the first systematic review to predict the risk of DKD in T2DM patients using ML algorithms. We categorized ML models according to their chronological development into traditional regression ML models, general ML models, and DL models, and subsequently compared their performance. Our results indicated that DL models consistently outperform both regression-based and other ML models, aligning with findings from previous studies (71, 72). The superior performance of DL models in these studies can be attributed to their capacity to process unstructured data (73). Such data include diabetic retinal images, renal ultrasound scans, and renal pathology slides, which directly reflect the pathological state of DKD. DL models (CNN, ANN et al) are capable of automatically extracting high-dimensional features such as vascular morphology and renal tissue microstructure, which are often challenging for other traditional ML techniques to capture (74). However, traditional ML algorithms can still achieve high performance with less computational power and smaller datasets. These models predominantly rely on structured data, including demographic information, laboratory metrics, lifestyle factors, and other clearly defined data with standardized units, which underscores their inherent strengths. Notably, the study by Betzler et al. found that modeling based on DL in combination with risk factors obtained by RF screening yielded more effective results. Consequently, future studies that can integrate structural data and unstructured data by multimodal modeling may further improve the prediction of DKD risk.

In addition, we evaluated and compared the performance of common ML models (e.g., LR, RF, DT, SVM, etc.) separately. Our results indicated that the RF model yielded the highest performance, with a pooled AUC of 0.848 (95% CI 0.785-0.911), outperforming the other models. This superior performance may be attributed to unique ability to its unique ability to handle feature importance, strong generalization, avoidance of overfitting and proficiency in processing high-dimensional data (75, 76). RF is an ensemble learning algorithm that solves classification and regression problems by constructing multiple DTs, enabling the model to effectively assess the relative importance of each feature in prediction (77). This characteristic aids in identifying the most influential predictors of DKD risk, thereby providing valuable support for clinical decision-making. Additionally, RF trains each tree by randomly selecting subsets of features and data, a strategy that significantly reduces the overfitting risks typically associated with individual models, thereby enhancing the model’s generalizability. Moreover, RF’s parallelization capability results in high computational efficiency, markedly reducing training time (78). This efficiency enables the RF model to process large-scale clinical data, such as electronic health records, genomic data, and imaging information, quickly. This makes RF particularly suitable for clinical settings where real-time predictions or immediate decision-making are required. Unlike our study, the meta-analysis by Feng et al. (79) found that the LR model achieved a higher AUC in predicting stroke onset timing. Similarly, Wu et al. reported that neural network algorithms outperformed others in screening for diabetic retinopathy (80). These results suggest that, in practical applications, the selection of a model should be based on the specific research objectives and the characteristics of the dataset.

The selection of effective features is of critical importance for the enhancement of both the predictive accuracy and interpretability of ML models in clinical applications. To this end, a comprehensive integration of the predictors that were ultimately incorporated into the models was conducted. The final selection comprised fourteen distinct categories of predictors, including demographic data, medical history, duration of T2DM, prior medication use, comorbidity profiles, lifestyle factors, family medical history, physical examination results, laboratory findings, renal biopsy data, radiomic features, circulating metabolites, genetic markers, and retinal imaging results. The heterogeneity of these predictive factors is reflective of the intricate and multifaceted pathophysiological underpinnings of DKD risk (81, 82). It is noteworthy that age has been consistently validated in numerous studies as a pivotal predictor of DKD (5). Moreover, the duration of T2DM is recognized as a pivotal factor in DKD progression. Extensive research indicates that a longer duration of T2DM significantly escalates the risk of DKD, a phenomenon that is intrinsically linked to the chronic renal damage induced by sustained hyperglycemia (83, 84). Furthermore, complications, such as diabetic retinopathy and peripheral vascular disease are also closely associated with an increased risk of DKD. These complications serve as hallmarks of systemic microvascular pathology, indicating an increased likelihood of renal impairment (85). The integration of emerging predictive factors, such as renal biopsy data, circulating metabolites, genetic markers, and radiomic features, underscores the forefront of interdisciplinary approaches in DKD risk prediction. These high-dimensional datasets enrich the input variables for complex models, thereby enhancing predictive precision and bolstering the models’ generalizability (81).

This study represents the first comprehensive review of the application of ML models for predicting DKD risk in patients with T2DM. A principal strength of our study is the rigorous and systematically developed methodology, which is not only logically sound but also sufficiently detailed to ensure reproducibility. Almost all published studies on the risk of DKD in patients with T2DM were included in this meta-analysis, facilitating comprehensive comparisons across different studies. In addition, we extensive searched multiple databases in accordance with PRISMA guidelines. Two independent reviewers systematically accomplished literature screening, data extraction, and a detailed risk of bias assessment using the PROBAST checklist, which improved the credibility and reliability of the study results.

The present study also reveals a number of limitations (1). The limited availability of external validation studies constrains the generalizability of our findings (2). The studies included in this meta-analysis varied in design, with the majority being retrospective single-center cohort studies, which are prone to selection bias and information bias (3). There is considerable variation in sample sizes across studies. Studies with smaller sample sizes may experience greater random error in estimating feature factors, leading to potential overfitting or underfitting of the model (4). The variability in the sources of predictive factors also affected the consistency of the study results. While the diversity of data sources enriches the model’s input dimensions, it may also introduce issues related to data quality and consistency (5). Most studies exhibit notable shortcomings in handling missing data. These discrepancies may affect the prediction accuracy and stability of the models (6). There is considerable variation in the methods used for selecting predictive factors, and the choice of method can influence both the complexity and interpretability of the model.

The integration of ML with medical expertise maximizes its clinical efficacy. By providing clinicians with patient risk information and encouraging the active use of clinical decision-making tools, rather than passive reliance on them, early detection, treatment, and prognosis of diseases are significantly enhanced. Additionally, the streamlined presentation of data improves clinicians’ knowledge and experience, while the application of ML models can substantially reduce both time and economic costs. Therefore, we recommend that future research focus on the following aspects to enhance the standardization of study conduct and reporting, thereby advancing the field (1): In terms of study design, future research should focus on conducting large-scale prospective studies. (2) Concerning study populations, it is crucial to select appropriate data sources and ensure the rationality of inclusion and exclusion criteria. (3) For predictive factors, it is important to clearly define and assess these factors consistently across all populations and verify the effectiveness of the factors included in predictive models. (4) Appropriate methods for handling missing data should be employed. (5) Future research should place greater emphasis on external validation, particularly through the use of multi-center, large-scale datasets across diverse regions, to enhance the generalizability of the models. (6) In the discussion of results, a detailed evaluation of potential biases should be provided, along with exploration of strategies to mitigate these biases. (7) Whenever feasible, research data and processing codes should be made publicly available, and standardized evaluation datasets should be established to enhance the reproducibility and generalizability of future research. (8) Future studies should emphasize the transparency and standardization of data processing methods to ensure the reliability and broader applicability of results. (9) The integration of interpretable algorithms, such as the Shapley additive explanation (SHAP) algorithm, to interpret risk prediction models can help clinicians better understand the key drivers behind model predictions, thereby enhancing clinical decision-making.

## 5 Conclusion

ML algorithms have demonstrated high performance in predicting the risk of DKD in T2DM patients. By integrating various demographic characteristics, biomarkers, and clinical data, these models offer more precise risk predictions compared to traditional methods. However, challenges related to data bias during model development and validation still need to be addressed. Future research should focus on enhancing data transparency and standardization, as well as validating these models’ generalizability through multicenter studies. In summary, the application of machine learning in predicting diabetic nephropathy risk holds significant clinical value and represents a more selective and cost-effective screening method for diabetic nephropathy.

## Data Availability

The original contributions presented in the study are included in the article/**Supplementary Material**. Further inquiries can be directed to the corresponding authors.

## References

[1] Kidney Disease: Improving Global Outcomes (KDIGO) Diabetes Work Group . KDIGO 2022 clinical practice guideline for diabetes management in chronic kidney disease. Kidney Int. (2022) 102:S1–s127. doi: 10.1016/j.kint.2022.06.008 36272764

[2] Sun H Saeedi P Karuranga S Pinkepank M Ogurtsova K Duncan BB . IDF Diabetes Atlas: Global, regional and country-level diabetes prevalence estimates for 2021 and projections for 2045. Diabetes Res Clin Pract. (2022) 183:109119. doi: 10.1016/j.diabres.2021.109119 34879977 PMC11057359

[3] American Diabetes Association . 11. Microvascular complications and foot care: standards of medical care in diabetes-2019. Diabetes Care. (2019) 42:S124–s38. doi: 10.2337/dc19-S011 30559237

[4] Chen J Zhang Q Liu D Liu Z . Exosomes: Advances, development and potential therapeutic strategies in diabetic nephropathy. Metabolism. (2021) 122:154834. doi: 10.1016/j.metabol.2021.154834 34217734

[5] Koye DN Magliano DJ Nelson RG Pavkov ME . The global epidemiology of diabetes and kidney disease. Adv Chronic Kidney Dis. (2018) 25:121–32. doi: 10.1053/j.ackd.2017.10.011 PMC11000253 29580576

[6] Thomas MC Brownlee M Susztak K Sharma K Jandeleit-Dahm KA Zoungas S . Diabetic kidney disease. Nat Rev Dis Primers. (2015) 1:15018. doi: 10.1038/nrdp.2015.18 27188921 PMC7724636

[7] Zhang XX Kong J Yun K . Prevalence of diabetic nephropathy among patients with type 2 diabetes mellitus in China: A meta-analysis of observational studies. J Diabetes Res. (2020) 2020:2315607. doi: 10.1155/2020/2315607 32090116 PMC7023800

[8] Saputro SA Pattanaprateep O Pattanateepapon A Karmacharya S Thakkinstian A . Prognostic models of diabetic microvascular complications: a systematic review and meta-analysis. Syst Rev. (2021) 10:288. doi: 10.1186/s13643-021-01841-z 34724973 PMC8561867

[9] Hippisley-Cox J Coupland C Robson J Sheikh A Brindle P . Predicting risk of type 2 diabetes in England and Wales: prospective derivation and validation of QDScore. Bmj. (2009) 338:b880. doi: 10.1136/bmj.b880 19297312 PMC2659857

[10] Noble D Mathur R Dent T Meads C Greenhalgh T . Risk models and scores for type 2 diabetes: systematic review. Bmj. (2011) 343:d7163. doi: 10.1136/bmj.d7163 22123912 PMC3225074

[11] Chen L Magliano DJ Zimmet PZ . The worldwide epidemiology of type 2 diabetes mellitus–present and future perspectives. Nat Rev Endocrinol. (2011) 8:228–36. doi: 10.1038/nrendo.2011.183 22064493

[12] Jacobs PG Herrero P Facchinetti A Vehi J Kovatchev B Breton MD . Artificial intelligence and machine learning for improving glycemic control in diabetes: best practices, pitfalls, and opportunities. IEEE Rev BioMed Eng. (2024) 17:19–41. doi: 10.1109/rbme.2023.3331297 37943654

[13] Rajkomar A Dean J Kohane I . Machine learning in medicine. N Engl J Med. (2019) 380:1347–58. doi: 10.1056/NEJMra1814259 30943338

[14] Obermeyer Z Emanuel EJ . Predicting the future - big data, machine learning, and clinical medicine. N Engl J Med. (2016) 375:1216–9. doi: 10.1056/NEJMp1606181 PMC5070532 27682033

[15] Tan H Shi Y Yue T Zheng D Luo S Weng J . Machine learning approach reveals microbiome, metabolome, and lipidome profiles in type 1 diabetes. J Adv Res. (2023) 64:213–21. doi: 10.1016/j.jare.2023.11.025 PMC11464464 38042287

[16] Esteva A Robicquet A Ramsundar B Kuleshov V DePristo M Chou K . A guide to deep learning in healthcare. Nat Med. (2019) 25:24–9. doi: 10.1038/s41591-018-0316-z 30617335

[17] Kim YK Na KS . Application of machine learning classification for structural brain MRI in mood disorders: Critical review from a clinical perspective. Prog Neuropsychopharmacol Biol Psychiatry. (2018) 80:71–80. doi: 10.1016/j.pnpbp.2017.06.024 28648568

[18] Slieker RC Münch M Donnelly LA Bouland GA Dragan I Kuznetsov D . An omics-based machine learning approach to predict diabetes progression: a RHAPSODY study. Diabetologia. (2024) 67:885–94. doi: 10.1007/s00125-024-06105-8 PMC10954972 38374450

[19] Jordan MI Mitchell TM . Machine learning: Trends, perspectives, and prospects. Science. (2015) 349:255–60. doi: 10.1126/science.aaa8415 26185243

[20] Persson F Rossing P . Diagnosis of diabetic kidney disease: state of the art and future perspective. Kidney Int Suppl (2011). (2018) 8:2–7. doi: 10.1016/j.kisu.2017.10.003 30675433 PMC6336222

[21] Colhoun HM Marcovecchio ML . Biomarkers of diabetic kidney disease. Diabetologia. (2018) 61:996–1011. doi: 10.1007/s00125-018-4567-5 29520581 PMC6448994

[22] Cole JB Florez JC . Genetics of diabetes mellitus and diabetes complications. Nat Rev Nephrol. (2020) 16:377–90. doi: 10.1038/s41581-020-0278-5 PMC9639302 32398868

[23] Yip W Ong PG Teo BW Cheung CY Tai ES Cheng CY . Retinal vascular imaging markers and incident chronic kidney disease: A prospective cohort study. Sci Rep. (2017) 7:9374. doi: 10.1038/s41598-017-09204-2 28839244 PMC5570935

[24] Eddy S Mariani LH Kretzler M . Integrated multi-omics approaches to improve classification of chronic kidney disease. Nat Rev Nephrol. (2020) 16:657–68. doi: 10.1038/s41581-020-0286-5 32424281

[25] Lekha S MS . Recent advancements and future prospects on E-nose sensors technology and machine learning approaches for non-invasive diabetes diagnosis: A review. IEEE Rev BioMed Eng. (2021) 14:127–38. doi: 10.1109/rbme.2020.2993591 32396102

[26] Sabanayagam C He F Nusinovici S Li J Lim C Tan G . Prediction of diabetic kidney disease risk using machine learning models: A population-based cohort study of Asian adults. Elife. (2023) 12:e81878. doi: 10.7554/eLife.81878 37706530 PMC10531395

[27] Nicolucci A Romeo L Bernardini M Vespasiani M Rossi MC Petrelli M . Prediction of complications of type 2 Diabetes: A Machine learning approach. Diabetes Res Clin Pract. (2022) 190:110013. doi: 10.1016/j.diabres.2022.110013 35870573

[28] Page MJ McKenzie JE Bossuyt PM Boutron I Hoffmann TC Mulrow CD . The PRISMA 2020 statement: an updated guideline for reporting systematic reviews. Bmj. (2021) 372:n71. doi: 10.1136/bmj.n71 33782057 PMC8005924

[29] Moons KG de Groot JA Bouwmeester W Vergouwe Y Mallett S Altman DG . Critical appraisal and data extraction for systematic reviews of prediction modelling studies: the CHARMS checklist. PloS Med. (2014) 11:e1001744. doi: 10.1371/journal.pmed.1001744 25314315 PMC4196729

[30] Canellas J Ritto FG Rodolico A Aguglia E Fernandes GVO Figueredo C . The international platform of registered systematic review and meta-analysis protocols (INPLASY) at 3 years: an analysis of 4,658 registered protocols on inplasy.com, platform features, and website statistics. Front Res Metr Anal. (2023) 8:1135853. doi: 10.3389/frma.2023.1135853 37588882 PMC10426905

[31] American Diabetes Association . 2. Classification and diagnosis of diabetes: standards of medical care in diabetes-2018. Diabetes Care. (2018) 41:S13–s27. doi: 10.2337/dc18-S002 29222373

[32] Collins GS Moons KGM Dhiman P Riley RD Beam AL Van Calster B . TRIPOD+AI statement: updated guidance for reporting clinical prediction models that use regression or machine learning methods. Bmj. (2024) 385:e078378. doi: 10.1136/bmj-2023-078378 38626948 PMC11019967

[33] Moons KGM Wolff RF Riley RD Whiting PF Westwood M Collins GS . PROBAST: A tool to assess risk of bias and applicability of prediction model studies: explanation and elaboration. Ann Intern Med. (2019) 170:W1–w33. doi: 10.7326/m18-1377 30596876

[34] Debray TP Damen JA Riley RD Snell K Reitsma JB Hooft L . A framework for meta-analysis of prediction model studies with binary and time-to-event outcomes. Stat Methods Med Res. (2019) 28:2768–86. doi: 10.1177/0962280218785504 PMC6728752 30032705

[35] Hanley JA McNeil BJ . The meaning and use of the area under a receiver operating characteristic (ROC) curve. Radiology. (1982) 143:29–36. doi: 10.1148/radiology.143.1.7063747 7063747

[36] Altman DG Bland JM . How to obtain the confidence interval from a P value. Bmj. (2011) 343:d2090. doi: 10.1136/bmj.d2090 21824904

[37] DerSimonian R Laird N . Meta-analysis in clinical trials revisited. Contemp Clin Trials. (2015) 45:139–45. doi: 10.1016/j.cct.2015.09.002 PMC4639420 26343745

[38] Thorlund K Wetterslev J Awad T Thabane L Gluud C . Comparison of statistical inferences from the DerSimonian-Laird and alternative random-effects model meta-analyses - an empirical assessment of 920 Cochrane primary outcome meta-analyses. Res Synth Methods. (2011) 2:238–53. doi: 10.1002/jrsm.53 26061888

[39] Zhao Y Liu L Zuo L Zhou X Wang S Gao H . A novel risk score model for the differential diagnosis of type 2 diabetic nephropathy: A multicenter study. J Diabetes Res. (2023) 2023:5514767. doi: 10.1155/2023/5514767 38155834 PMC10754636

[40] Liu S Zhang R Shang X Li W . Analysis for warning factors of type 2 diabetes mellitus complications with Markov blanket based on a Bayesian network model. Comput Methods Programs Biomed. (2020) 188:105302. doi: 10.1016/j.cmpb.2019.105302 31923820

[41] Hui D Sun Y Xu S Liu J He P Deng Y . Analysis of clinical predictors of kidney diseases in type 2 diabetes patients based on machine learning. Int Urol Nephrol. (2023) 55:687–96. doi: 10.1007/s11255-022-03322-1 36069963

[42] Momenzadeh A Shamsa A Meyer JG . Bias or biology? Importance of model interpretation in machine learning studies from electronic health records. JAMIA Open. (2022) 5:ooac063. doi: 10.1093/jamiaopen/ooac063 35958671 PMC9360778

[43] Cai SS Zheng TY Wang KY Zhu HP . Clinical study of different prediction models in predicting diabetic nephropathy in patients with type 2 diabetes mellitus. World J Diabetes. (2024) 15:43–52. doi: 10.4239/wjd.v15.i1.43 38313855 PMC10835501

[44] Betzler BK Chee EYL He F Lim CC Ho J Hamzah H . Deep learning algorithms to detect diabetic kidney disease from retinal photographs in multiethnic populations with diabetes. J Am Med Inform Assoc. (2023) 30:1904–14. doi: 10.1093/jamia/ocad179 PMC10654858 37659103

[45] He F Ng Yin Ling C Nusinovici S Cheng CY Wong TY Li J . Development and external validation of machine learning models for diabetic microvascular complications: cross-sectional study with metabolites. J Med Internet Res. (2024) 26:e41065. doi: 10.2196/41065 38546730 PMC11009843

[46] Hosseini Sarkhosh SM Hemmatabadi M Esteghamati A . Development and validation of a risk score for diabetic kidney disease prediction in type 2 diabetes patients: a machine learning approach. J Endocrinol Invest. (2023) 46:415–23. doi: 10.1007/s40618-022-01919-y 36114952

[47] Afrash MR Rahimi F Kazemi-Arpanahi H Shanbezadeh M Amraei M Asadi F . Development of an intelligent clinical decision support system for the early prediction of diabetic nephropathy. Inf Med Unlocked. (2022) 35:101135. doi: 10.1016/j.imu.2022.101135

[48] Yang D Tian C Liu J Peng Y Xiong Z Da J . Diffusion tensor and kurtosis MRI-based radiomics analysis of kidney injury in type 2 diabetes. J Magn Reson Imaging. (2024) 60(5):2078. doi: 10.1002/jmri.29263 38299753

[49] Yin JM Li Y Xue JT Zong GW Fang ZZ Zou L . Explainable machine learning-based prediction model for diabetic nephropathy. J Diabetes Res. (2024) 2024:8857453. doi: 10.1155/2024/8857453 38282659 PMC10821806

[50] Song X Waitman LR Yu AS Robbins DC Hu Y Liu M . Longitudinal risk prediction of chronic kidney disease in diabetic patients using a temporal-enhanced gradient boosting machine: retrospective cohort study. JMIR Med Inform. (2020) 8:e15510. doi: 10.2196/15510 32012067 PMC7055762

[51] Fan Y Long E Cai L Cao Q Wu X Tong R . Machine learning approaches to predict risks of diabetic complications and poor glycemic control in nonadherent type 2 diabetes. Front Pharmacol. (2021) 12:665951. doi: 10.3389/fphar.2021.665951 34239440 PMC8258097

[52] Zhang W Liu X Dong Z Wang Q Pei Z Chen Y . New diagnostic model for the differentiation of diabetic nephropathy from non-diabetic nephropathy in chinese patients. Front Endocrinol (Lausanne). (2022) 13:913021. doi: 10.3389/fendo.2022.913021 35846333 PMC9279696

[53] Liu XZ Duan M Huang HD Zhang Y Xiang TY Niu WC . Predicting diabetic kidney disease for type 2 diabetes mellitus by machine learning in the real world: a multicenter retrospective study. Front Endocrinol (Lausanne). (2023) 14:1184190. doi: 10.3389/fendo.2023.1184190 37469989 PMC10352831

[54] Hosseini Sarkhosh SM Esteghamati A Hemmatabadi M Daraei M . Predicting diabetic nephropathy in type 2 diabetic patients using machine learning algorithms. J Diabetes Metab Disord. (2022) 21:1433–41. doi: 10.1007/s40200-022-01076-2 PMC9672147 36404838

[55] Dong Z Wang Q Ke Y Zhang W Hong Q Liu C . Prediction of 3-year risk of diabetic kidney disease using machine learning based on electronic medical records. J Transl Med. (2022) 20:143. doi: 10.1186/s12967-022-03339-1 35346252 PMC8959559

[56] Allen A Iqbal Z Green-Saxena A Hurtado M Hoffman J Mao Q . Prediction of diabetic kidney disease with machine learning algorithms, upon the initial diagnosis of type 2 diabetes mellitus. BMJ Open Diabetes Res Care. (2022) 10:e002560. doi: 10.1136/bmjdrc-2021-002560 PMC8772425 35046014

[57] Maniruzzaman M Islam MM Rahman MJ Hasan MAM Shin J . Risk prediction of diabetic nephropathy using machine learning techniques: A pilot study with secondary data. Diabetes Metab Syndr. (2021) 15:102263. doi: 10.1016/j.dsx.2021.102263 34482122

[58] Song X Waitman LR Hu Y Yu ASL Robbins DC Liu M . Robust clinical marker identification for diabetic kidney disease with ensemble feature selection. J Am Med Inform Assoc. (2019) 26:242–53. doi: 10.1093/jamia/ocy165 PMC7792755 30602020

[59] Wang G Wang B Qiao G Lou H Xu F Chen Z . Screening tools based on nomogram for diabetic kidney diseases in chinese type 2 diabetes mellitus patients. Diabetes Metab J. (2021) 45:708–18. doi: 10.4093/dmj.2020.0117 PMC8497917 33844903

[60] Shi S Gao L Zhang J Zhang B Xiao J Xu W . The automatic detection of diabetic kidney disease from retinal vascular parameters combined with clinical variables using artificial intelligence in type-2 diabetes patients. BMC Med Inform Decis Mak. (2023) 23:241. doi: 10.1186/s12911-023-02343-9 37904184 PMC10617171

[61] Leung RK Wang Y Ma RC Luk AO Lam V Ng M . Using a multi-staged strategy based on machine learning and mathematical modeling to predict genotype-phenotype risk patterns in diabetic kidney disease: a prospective case-control cohort analysis. BMC Nephrol. (2013) 14:162. doi: 10.1186/1471-2369-14-162 23879411 PMC3726338

[62] Su X Lin S Huang Y . Value of radiomics-based two-dimensional ultrasound for diagnosing early diabetic nephropathy. Sci Rep. (2023) 13:20427. doi: 10.1038/s41598-023-47449-2 37993534 PMC10665410

[63] Levin A Stevens PE . Early detection of CKD: the benefits, limitations and effects on prognosis. Nat Rev Nephrol. (2011) 7:446–57. doi: 10.1038/nrneph.2011.86 21712852

[64] Collins GS de Groot JA Dutton S Omar O Shanyinde M Tajar A . External validation of multivariable prediction models: a systematic review of methodological conduct and reporting. BMC Med Res Methodol. (2014) 14:40. doi: 10.1186/1471-2288-14-40 24645774 PMC3999945

[65] Steyerberg EW Moons KG van der Windt DA Hayden JA Perel P Schroter S . Prognosis Research Strategy (PROGRESS) 3: prognostic model research. PloS Med. (2013) 10:e1001381. doi: 10.1371/journal.pmed.1001381 23393430 PMC3564751

[66] Siontis GC Tzoulaki I Castaldi PJ Ioannidis JP . External validation of new risk prediction models is infrequent and reveals worse prognostic discrimination. J Clin Epidemiol. (2015) 68:25–34. doi: 10.1016/j.jclinepi.2014.09.007 25441703

[67] Bates DW Auerbach A Schulam P Wright A Saria S . Reporting and implementing interventions involving machine learning and artificial intelligence. Ann Intern Med. (2020) 172:S137–s44. doi: 10.7326/m19-0872 32479180

[68] Moons KG Kengne AP Grobbee DE Royston P Vergouwe Y Altman DG . Risk prediction models: II. External validation, model updating, and impact assessment. Heart. (2012) 98:691–8. doi: 10.1136/heartjnl-2011-301247 22397946

[69] la-Roi-Teeuw HM van Royen FS de Hond A Zahra A de Vries S Bartels R . Don't be misled: 3 misconceptions about external validation of clinical prediction models. J Clin Epidemiol. (2024) 172:111387. doi: 10.1016/j.jclinepi.2024.111387 38729274

[70] Riley RD Archer L Snell KIE Ensor J Dhiman P Martin GP . Evaluation of clinical prediction models (part 2): how to undertake an external validation study. Bmj. (2024) 384:e074820. doi: 10.1136/bmj-2023-074820 38224968 PMC10788734

[71] Smith LA Oakden-Rayner L Bird A Zeng M To MS Mukherjee S . Machine learning and deep learning predictive models for long-term prognosis in patients with chronic obstructive pulmonary disease: a systematic review and meta-analysis. Lancet Digit Health. (2023) 5:e872–e81. doi: 10.1016/s2589-7500(23)00177-2 38000872

[72] Tu JV . Advantages and disadvantages of using artificial neural networks versus logistic regression for predicting medical outcomes. J Clin Epidemiol. (1996) 49:1225–31. doi: 10.1016/s0895-4356(96)00002-9 8892489

[73] LeCun Y Bengio Y Hinton G . Deep learning. Nature. (2015) 521:436–44. doi: 10.1038/nature14539 26017442

[74] Fogo AB . Learning from deep learning and pathomics. Kidney Int. (2023) 104:1050–3. doi: 10.1016/j.kint.2023.06.006 37336291

[75] Liu J Wang H Hang H Ma S Shen X Shi Y . Self-supervised random forest on transformed distribution for anomaly detection. IEEE Trans Neural Netw Learn Syst. (2024) 36:2675–89. doi: 10.1109/tnnls.2023.3348833 38261504

[76] Breiman L . Manual on setting up, using, and understanding random forests v3. 1 Vol. 1. Statistics Department University of California Berkeley, CA, USA (2002) p. 3–42.

[77] Speiser JL Miller ME Tooze J Ip E . A comparison of random forest variable selection methods for classification prediction modeling. Expert Syst Appl. (2019) 134:93–101. doi: 10.1016/j.eswa.2019.05.028 32968335 PMC7508310

[78] Paul A Mukherjee DP Das P Gangopadhyay A Chintha AR Kundu S . Improved random forest for classification. IEEE Trans Image Process. (2018) 27:4012–24. doi: 10.1109/tip.2018.2834830 29993742

[79] Feng J Zhang Q Wu F Peng J Li Z Chen Z . The value of applying machine learning in predicting the time of symptom onset in stroke patients: systematic review and meta-analysis. J Med Internet Res. (2023) 25:e44895. doi: 10.2196/44895 37824198 PMC10603565

[80] Wu JH Liu TYA Hsu WT Ho JH Lee CC . Performance and limitation of machine learning algorithms for diabetic retinopathy screening: meta-analysis. J Med Internet Res. (2021) 23:e23863. doi: 10.2196/23863 34407500 PMC8406115

[81] Alicic RZ Rooney MT Tuttle KR . Diabetic kidney disease: challenges, progress, and possibilities. Clin J Am Soc Nephrol. (2017) 12:2032–45. doi: 10.2215/cjn.11491116 PMC5718284 28522654

[82] Pugliese G Penno G Natali A Barutta F Di Paolo S Reboldi G . Diabetic kidney disease: New clinical and therapeutic issues. Joint position statement of the Italian Diabetes Society and the Italian Society of Nephrology on "The natural history of diabetic kidney disease and treatment of hyperglycemia in patients with type 2 diabetes and impaired renal function. Nutr Metab Cardiovasc Dis. (2019) 29:1127–50. doi: 10.1016/j.numecd.2019.07.017 31586514

[83] Wang G Ouyang J Li S Wang H Lian B Liu Z . The analysis of risk factors for diabetic nephropathy progression and the construction of a prognostic database for chronic kidney diseases. J Transl Med. (2019) 17:264. doi: 10.1186/s12967-019-2016-y 31409386 PMC6693179

[84] Siddiqui K George TP Joy SS Alfadda AA . Risk factors of chronic kidney disease among type 2 diabetic patients with longer duration of diabetes. Front Endocrinol (Lausanne). (2022) 13:1079725. doi: 10.3389/fendo.2022.1079725 36568108 PMC9780388

[85] Jha R Lopez-Trevino S Kankanamalage HR Jha JC . Diabetes and renal complications: an overview on pathophysiology, biomarkers and therapeutic interventions. Biomedicines. (2024) 12:1098. doi: 10.3390/biomedicines12051098 38791060 PMC11118045

