# Supplementary material for: Machine learning-based risk predictive models for diabetic kidney disease in type 2 diabetes mellitus patients: a systematic review and meta-analysis
Source: Front Endocrinol (Lausanne). 2025 Mar 3;16:1495306. doi: 10.3389/fendo.2025.1495306 (PMC11911190; doi:10.3389/fendo.2025.1495306)
Supplement: Supplementary file 1 [file DataSheet1.docx]

Supplementary Material

# Supplementary Tables and Figures

## Supplementary Tables

**Supplemental Table 1A Detailed search strategy of PubMed**

| **Search** | **Query** | **Items found** |
| --- | --- | --- |
| #1 | Search: "Diabetes Mellitus, Type 2"[Mesh] | 179012 |
| #2 | Search: ((((((((((((((((((((((((((((Diabetes Mellitus, Noninsulin-Dependent[Title/Abstract]) OR (Diabetes Mellitus, Ketosis-Resistant[Title/Abstract])) OR (Diabetes Mellitus, Ketosis Resistant[Title/Abstract])) OR (Ketosis-Resistant Diabetes Mellitus[Title/Abstract])) OR (Diabetes Mellitus, Non Insulin Dependent[Title/Abstract])) OR (Diabetes Mellitus, Non-Insulin-Dependent[Title/Abstract])) OR (Non-Insulin-Dependent Diabetes Mellitus[Title/Abstract])) OR (Diabetes Mellitus, Stable[Title/Abstract])) OR (Stable Diabetes Mellitus[Title/Abstract])) OR (Diabetes Mellitus, Type II[Title/Abstract])) OR (Diabetes Mellitus, Noninsulin Dependent[Title/Abstract])) OR (Diabetes Mellitus, Maturity-Onset[Title/Abstract])) OR (Diabetes Mellitus, Maturity Onset[Title/Abstract])) OR (Maturity-Onset Diabetes Mellitus[Title/Abstract])) OR (Maturity-Onset Diabetes Mellitus[Title/Abstract])) OR (Diabetes Mellitus, Slow-Onset[Title/Abstract])) OR (Diabetes Mellitus, Slow Onset[Title/Abstract])) OR (Slow-Onset Diabetes Mellitus[Title/Abstract])) OR (Type 2 Diabetes Mellitus[Title/Abstract])) OR (Noninsulin-Dependent Diabetes Mellitus[Title/Abstract])) OR (Noninsulin Dependent Diabetes Mellitus[Title/Abstract])) OR (Maturity-Onset Diabetes[Title/Abstract])) OR (Diabetes, Maturity-Onset[Title/Abstract])) OR (Maturity Onset Diabetes[Title/Abstract])) OR (Type 2 Diabetes[Title/Abstract])) OR (Diabetes, Type 2[Title/Abstract])) OR (Diabetes Mellitus, Adult-Onset[Title/Abstract])) OR (Adult-Onset Diabetes Mellitus[Title/Abstract])) OR (Diabetes Mellitus, Adult Onset[Title/Abstract]) | 187664 |
| #3 | Search: (#1) OR (#2) | 243363 |
| #4 | Search: "Machine Learning"[Mesh] | 66517 |
| #5 | Search: ((((((((((((((((((((((((((((Learning, Machine[Title/Abstract]) OR (Transfer Learning[Title/Abstract])) OR (Learning, Transfer[Title/Abstract])) OR (Supervised Machine Learning[Title/Abstract])) OR (Linear regression[Title/Abstract])) OR (Logistic regression[Title/Abstract])) OR (Decision trees[Title/Abstract])) OR (K-Nearest Neighbors[Title/Abstract])) OR (Liner discriminant analysis[Title/Abstract])) OR (Support Vector Machine[Title/Abstract])) OR (Naive bayes[Title/Abstract])) OR (Random forest[Title/Abstract])) OR (Adaptive boosting[Title/Abstract])) OR (Gradient boosting[Title/Abstract])) OR (Unsupervised Machine Learning[Title/Abstract])) OR (K-Means Clustering[Title/Abstract])) OR (Principal component analysis[Title/Abstract])) OR (Gaussian mixture models[Title/Abstract])) OR (Hierarchical clustering[Title/Abstract])) OR (Autoencoders[Title/Abstract])) OR (deep learning[Title/Abstract])) OR (Convolutional neural networks[Title/Abstract])) OR (Recurrent neural networks[Title/Abstract])) OR (Transformer[Title/Abstract])) OR (Feedforward neural networks[Title/Abstract])) OR (Long short-term memory[Title/Abstract])) OR (Reinforcement learning[Title/Abstract])) OR (Q-Learning[Title/Abstract])) OR (Deep Reinforcement learning[Title/Abstract]) | 753747 |
| #6 | Search: (#4) OR (#5) | 778571 |
| #7 | Search: "Diabetic Nephropathies"[Mesh] | 30356 |
| #8 | Search: ((((((((Nephropathies, Diabetic[Title/Abstract]) OR (Nephropathy, Diabetic[Title/Abstract])) OR (Diabetic Nephropathy[Title/Abstract])) OR (Diabetic Kidney Disease[Title/Abstract])) OR (Diabetic Kidney Diseases[Title/Abstract])) OR (Kidney Disease, Diabetic[Title/Abstract])) OR (Kidney Diseases, Diabetic[Title/Abstract])) OR (Diabetic Glomerulosclerosis[Title/Abstract])) OR (Glomerulosclerosis, Diabetic[Title/Abstract]) | 28383 |
| #9 | Search: (#7) OR (#8) | 41165 |
| #10 | Search: ((((((((((((((((((Risk[Title/Abstract]) OR (Predict[Title/Abstract])) OR (Prediction[Title/Abstract])) OR (Forecast[Title/Abstract])) OR (Prognose[Title/Abstract])) OR (Prognosis[Title/Abstract])) OR (Prognostic[Title/Abstract])) OR (Assess[Title/Abstract])) OR (Assessment[Title/Abstract])) OR (Evaluate[Title/Abstract])) OR (Evaluation[Title/Abstract])) OR (Estimate[Title/Abstract])) OR (Calculate[Title/Abstract])) OR (Diagnose[Title/Abstract])) OR (Diagnosis[Title/Abstract])) OR (Diagnostic[Title/Abstract])) AND (model[Title/Abstract])) OR (Area under the curve[Title/Abstract])) OR (Area under the receiver operator curve[Title/Abstract]) | 1180569 |
| #11 | Search: (((#3) AND (#6)) AND (#9)) AND (#10) | 218 |

**Supplemental Table 1B Detailed search strategy of embase**

| **Search** | **Query** | **Items found** |
| --- | --- | --- |
| #1 | 'non insulin dependent diabetes mellitus'/exp OR 'adult onset diabetes':ti,ab OR 'adult onset diabetes mellitus':ti,ab OR 'diabetes mellitus type 2':ti,ab OR 'diabetes mellitus type ii':ti,ab OR 'diabetes mellitus, maturity onset':ti,ab OR 'diabetes mellitus, non insulin dependent':ti,ab OR 'diabetes mellitus, non-insulin-dependent':ti,ab OR 'diabetes mellitus, type 2':ti,ab OR 'diabetes mellitus, type ii':ti,ab OR 'diabetes type 2':ti,ab OR 'diabetes type ii':ti,ab OR 'diabetes, adult onset':ti,ab OR 'dm 2':ti,ab OR 'insulin independent diabetes':ti,ab OR 'insulin independent diabetes mellitus':ti,ab OR 'ketosis resistant diabetes mellitus':ti,ab OR 'maturity onset diabetes':ti,ab OR 'maturity onset diabetes mellitus':ti,ab OR 'niddm':ti,ab OR 'niddm (non insulin dependent diabetes mellitus)':ti,ab OR 'non insulin dependent (type 2) diabetes mellitus':ti,ab OR 'non insulin dependent diabetes':ti,ab OR 'non-insulin-dependent diabetes mellitu':ti,ab OR 'noninsulin dependent (type 2) diabetes mellitus':ti,ab OR 'noninsulin dependent diabetes':ti,ab OR 'noninsulin dependent diabetes mellitus':ti,ab OR 't2dm':ti,ab OR 'tiidm':ti,ab OR 'type 2 (insulin independent) diabetes':ti,ab OR 'type 2 diabetes':ti,ab OR 'type 2 diabetes mellitus':ti,ab OR 'type ii diabetes':ti,ab OR 'type ii diabetes mellitus':ti,ab OR 'non insulin dependent diabetes mellitus':ti,ab | 415179 |
| #2 | 'machine learning'/exp OR 'learning machine':ti,ab OR 'learning machines':ti,ab OR 'machine learning':ti,ab OR 'semi supervised machine learning':ti,ab OR 'supervised machine learning':ti,ab 'unsupervised machine learning':ti,ab OR 'linear regression':ti,ab OR 'logistic regression':ti,ab OR 'decision trees':ti,ab OR 'k-nearest neighbors':ti,ab OR 'liner discriminant analysis':ti,ab OR 'support vector machine':ti,ab OR 'bayesian learning':ti,ab OR 'random forest':ti,ab OR 'adaptive boosting':ti,ab OR 'gradient boosting':ti,ab OR 'k-means clustering':ti,ab OR 'principal component analysis':ti,ab OR 'gaussian mixture models':ti,ab OR 'hierarchical clustering':ti,ab OR 'autoencoders':ti,ab OR 'deep learning':ti,ab OR 'convolutional neural networks':ti,ab OR 'recurrent neural networks':ti,ab OR 'transformer':ti,ab OR 'feedforward neural networks':ti,ab OR 'long short-term memory':ti,ab OR 'reinforcement learning':ti,ab OR 'q-learning':ti,ab OR 'deep reinforcement learning':ti,ab OR 'artificial neural network':ti,ab | 1368618 |
| #3 | 'diabetic nephropathy'/exp OR 'nephropathies, diabetic':ti,ab OR 'nephropathy, diabetic':ti,ab OR 'diabetic nephropathy':ti,ab OR 'diabetic kidney disease':ti,ab OR 'diabetic kidney diseases':ti,ab OR 'kidney disease, diabetic':ti,ab OR 'kidney diseases, diabetic':ti,ab OR 'diabetic glomerulosclerosis':ti,ab OR 'glomerulosclerosis, diabetic':ti,ab | 64410 |
| #4 | 'risk':ti,ab OR 'predict':ti,ab OR 'prediction':ti,ab OR 'forecast':ti,ab OR 'prognose':ti,ab OR 'prognosis':ti,ab OR 'prognostic':ti,ab OR 'assess':ti,ab OR 'assessment':ti,ab OR 'evaluate':ti,ab OR 'evaluation':ti,ab OR 'estimate':ti,ab OR 'calculate':ti,ab OR 'diagnose':ti,ab OR 'diagnosis':ti,ab OR 'diagnostic':ti,ab OR area under the curve':ti,ab OR 'area under the receiver operator curve':ti,ab OR 'auc':ti,ab AND 'model':ti,ab | 1539471 |
| #4 | #1 AND #2 AND #3 AND #4 | 374 |

**Supplemental Table 1C Detailed search strategy of Web of Science**

| **Search** | **Query** | **Items found** |
| --- | --- | --- |
| #1 | (((((((((((((((((((((((((((((TS=(Diabetes Mellitus, Type 2)) OR TS=(Diabetes Mellitus, Noninsulin-Dependent)) OR TS=(Diabetes Mellitus, Ketosis-Resistant)) OR TS=(Diabetes Mellitus, Ketosis Resistant)) OR TS=(Ketosis-Resistant Diabetes Mellitus)) OR TS=(Diabetes Mellitus, Non Insulin Dependent)) OR TS=(Diabetes Mellitus, Non-Insulin-Dependent)) OR TS=(Non-Insulin-Dependent Diabetes Mellitus)) OR TS=(Diabetes Mellitus, Stable)) OR TS=(Stable Diabetes Mellitus)) OR TS=(Diabetes Mellitus, Type II)) OR TS=(Diabetes Mellitus, Noninsulin Dependent)) OR TS=(Diabetes Mellitus, Maturity-Onset)) OR TS=(Diabetes Mellitus, Maturity Onset)) OR TS=(Maturity-Onset Diabetes Mellitus)) OR TS=(Maturity Onset Diabetes Mellitus)) OR TS=(Diabetes Mellitus, Slow-Onset)) OR TS=(Diabetes Mellitus, Slow Onset)) OR TS=(Slow-Onset Diabetes Mellitus)) OR TS=(Type 2 Diabetes Mellitus)) OR TS=(Noninsulin-Dependent Diabetes Mellitus)) OR TS=(Noninsulin Dependent Diabetes Mellitus)) OR TS=(Maturity-Onset Diabetes)) OR TS=(Diabetes, Maturity-Onset)) OR TS=(Maturity Onset Diabetes)) OR TS=(Type 2 Diabetes)) OR TS=(Diabetes, Type 2)) OR TS=(Diabetes Mellitus, Adult-Onset)) OR TS=(Adult-Onset Diabetes Mellitus)) OR TS=(Diabetes Mellitus, Adult Onset) | 298328 |
| #2 | (((((((((((((((((((((((((((((TS=(Machine Learning)) OR TS=(Learning, Machine)) OR TS=(Transfer Learning)) OR TS=(Learning, Transfer)) OR TS=(Supervised Machine Learning)) OR TS=(Linear regression)) OR TS=(Logistic regression)) OR TS=(Decision trees)) OR TS=(K-Nearest Neighbors)) OR TS=(Liner discriminant analysis)) OR TS=(Support Vector Machine)) OR TS=(Naive bayes)) OR TS=(Random forest)) OR TS=(Adaptive boosting)) OR TS=(Gradient boosting)) OR TS=(Unsupervised Machine Learning)) OR TS=(K-Means Clustering)) OR TS=(Principal component analysis)) OR TS=(Gaussian mixture models)) OR TS=(Hierarchical clustering)) OR TS=(Autoencoders)) OR TS=(deep learning)) OR TS=(Convolutional neural networks)) OR TS=(Recurrent neural networks)) OR TS=(Transformer)) OR TS=(Feedforward neural networks)) OR TS=(Long short-term memory)) OR TS=(Reinforcement learning)) OR TS=(Q-Learning)) OR TS=(Deep Reinforcement learning) | 2106832 |
| #3 | (((((((((TS=(Diabetic Nephropathies)) OR TS=(Nephropathies, Diabetic)) OR TS=(Nephropathy, Diabetic)) OR TS=(Diabetic Nephropathy)) OR TS=(Diabetic Kidney Disease)) OR TS=(Diabetic Kidney Diseases)) OR TS=(Kidney Disease, Diabetic)) OR TS=(Kidney Diseases, Diabetic)) OR TS=(Diabetic Glomerulosclerosis)) OR TS=(Glomerulosclerosis, Diabetic) | 49909 |
| #4 | (((((((((((((((((TS=(Risk)) OR TS=(Predict)) OR TS=(Prediction)) OR TS=(Forecast)) OR TS=(Prognose)) OR TS=(Prognosis)) OR TS=(Prognostic)) OR TS=(Assess)) OR TS=(Assessment)) OR TS=(Evaluate)) OR TS=(Calculate)) OR TS=(Diagnose)) OR TS=(Diagnosis)) OR TS=(Diagnostic)) OR TS=(Area under the receiver operator curve)) OR TS=(area under the curve)) OR TS=(AUC)) AND TS=(model) | 5081236 |
| #4 | #1 AND #2 AND #3 AND #4 | 494 |

**Supplemental Table 1D Detailed search strategy of Cochrane Library**

| **Search** | **Query** | **Items found** |
| --- | --- | --- |
| #1 | ( ( TITLE-ABS-KEY ( diabetes AND mellitus, AND type 2 ) OR TITLE-ABS-KEY ( diabetes AND mellitus, AND noninsulin-dependent ) OR TITLE-ABS-KEY ( diabetes AND mellitus, AND ketosis-resistant ) OR TITLE-ABS-KEY ( diabetes AND mellitus, AND ketosis AND resistant ) OR TITLE-ABS-KEY ( ketosis-resistant AND diabetes AND mellitus ) OR TITLE-ABS-KEY ( diabetes AND mellitus, AND non AND insulin AND dependent ) OR TITLE-ABS-KEY ( diabetes AND mellitus, AND non-insulin-dependent ) OR TITLE-ABS-KEY ( non-insulin-dependent AND diabetes AND mellitus ) OR TITLE-ABS-KEY ( diabetes AND mellitus, AND stable ) OR TITLE-ABS-KEY ( stable AND diabetes AND mellitus ) OR TITLE-ABS-KEY ( diabetes AND mellitus, AND type AND ii ) OR TITLE-ABS-KEY ( diabetes AND mellitus, AND noninsulin AND dependent ) OR TITLE-ABS-KEY ( diabetes AND mellitus, AND maturity-onset ) OR TITLE-ABS-KEY ( diabetes AND mellitus, AND maturity AND onset ) OR TITLE-ABS-KEY ( maturity-onset AND diabetes AND mellitus ) OR TITLE-ABS-KEY ( maturity AND onset AND diabetes AND mellitus ) OR TITLE-ABS-KEY ( diabetes AND mellitus, AND slow-onset ) OR TITLE-ABS-KEY ( diabetes AND mellitus, AND slow AND onset ) OR TITLE-ABS-KEY ( slow-onset AND diabetes AND mellitus ) OR TITLE-ABS-KEY ( type 2 diabetes AND mellitus ) OR TITLE-ABS-KEY ( noninsulin-dependent AND diabetes AND mellitus ) OR TITLE-ABS-KEY ( noninsulin AND dependent AND diabetes AND mellitus ) OR TITLE-ABS-KEY ( maturity-onset AND diabetes ) OR TITLE-ABS-KEY ( diabetes, AND maturity-onset ) OR TITLE-ABS-KEY ( maturity AND onset AND diabetes ) OR TITLE-ABS-KEY ( type 2 diabetes ) OR TITLE-ABS-KEY ( diabetes, AND type 2 ) OR TITLE-ABS-KEY ( diabetes AND mellitus, AND adult-onset ) OR TITLE-ABS-KEY ( adult-onset AND diabetes AND mellitus ) OR TITLE-ABS-KEY ( diabetes AND mellitus, AND adult AND onset ) ) ) | 438061 |
| #2 | ( ( TITLE-ABS-KEY ( machine AND learning ) OR TITLE-ABS-KEY ( learning, AND machine ) OR TITLE-ABS-KEY ( transfer AND learning ) OR TITLE-ABS-KEY ( learning, AND transfer ) OR TITLE-ABS-KEY ( learning, AND transfer ) OR TITLE-ABS-KEY ( supervised AND machine AND learning ) OR TITLE-ABS-KEY ( linear AND regression ) OR TITLE-ABS-KEY ( logistic AND regression ) OR TITLE-ABS-KEY ( decision AND trees ) OR TITLE-ABS-KEY ( k-nearest AND neighbors ) OR TITLE-ABS-KEY ( liner AND discriminant AND analysis ) OR TITLE-ABS-KEY ( support AND vector AND machine ) OR TITLE-ABS-KEY ( naive AND bayes ) OR TITLE-ABS-KEY ( random AND forest ) OR TITLE-ABS-KEY ( adaptive AND boosting ) OR TITLE-ABS-KEY ( gradient AND boosting ) OR TITLE-ABS-KEY ( unsupervised AND machine AND learning ) OR TITLE-ABS-KEY ( k-means AND clustering ) OR TITLE-ABS-KEY ( principal AND component AND analysis ) OR TITLE-ABS-KEY ( gaussian AND mixture AND models ) OR TITLE-ABS-KEY ( hierarchical AND clustering ) OR TITLE-ABS-KEY ( autoencoders ) OR TITLE-ABS-KEY ( deep AND learning ) OR TITLE-ABS-KEY ( convolutional AND neural AND networks ) OR TITLE-ABS-KEY ( recurrent AND neural AND networks ) OR TITLE-ABS-KEY ( transformer ) OR TITLE-ABS-KEY ( feedforward AND neural AND networks ) OR TITLE-ABS-KEY ( long AND short-term AND memory ) OR TITLE-ABS-KEY ( reinforcement AND learning ) OR TITLE-ABS-KEY ( q-learning ) OR TITLE-ABS-KEY ( deep AND reinforcement AND learning ) ) ) | 2922825 |
| #3 | ( ( TITLE-ABS-KEY ( diabetic AND nephropathies ) OR TITLE-ABS-KEY ( nephropathies, AND diabetic ) OR TITLE-ABS-KEY ( nephropathy, AND diabetic ) OR TITLE-ABS-KEY ( diabetic AND nephropathy ) OR TITLE-ABS-KEY ( diabetic AND kidney AND disease ) OR TITLE-ABS-KEY ( diabetic AND kidney AND diseases ) OR TITLE-ABS-KEY ( kidney AND disease, AND diabetic ) OR TITLE-ABS-KEY ( kidney AND diseases, AND diabetic ) OR TITLE-ABS-KEY ( diabetic AND glomerulosclerosis ) OR TITLE-ABS-KEY ( glomerulosclerosis, AND diabetic ) ) ) | 83284 |
| #4 | ( ( TITLE-ABS-KEY ( risk ) OR TITLE-ABS-KEY ( predic ) OR TITLE-ABS-KEY ( prediction ) OR TITLE-ABS-KEY ( forecast ) OR TITLE-ABS-KEY ( prognose ) OR TITLE-ABS-KEY ( prognosis ) OR TITLE-ABS-KEY ( prognostic ) OR TITLE-ABS-KEY ( assess ) OR TITLE-ABS-KEY ( assessment ) OR TITLE-ABS-KEY ( evaluate ) OR TITLE-ABS-KEY ( calculate ) OR TITLE-ABS-KEY ( diagnose ) OR TITLE-ABS-KEY ( diagnosis ) OR TITLE-ABS-KEY ( diagnostic ) OR TITLE-ABS-KEY ( area AND under AND the AND receiver AND operator AND curve ) OR TITLE-ABS-KEY ( area AND under AND the AND curve ) OR TITLE-ABS-KEY ( auc ) ) ) AND ( TITLE-ABS-KEY ( model ) ) | 4583201 |
| #4 | #1 AND #2 AND #3 AND #4 | 174 |

**Supplemental Table 2 Risk of bias for each study using PROBAST Checklist**

| **Study** | **1-1** | **1-2** | **2-1** | **2-2** | **2-3** | **3-1** | **3-2** | **3-3** | **3-4** | **3-5** | **3-6** | **4-1** | **4-2** | **4-3** | **4-4** | **4-5** | **4-6** | **4-7** | **4-8** | **4-9** | **Risk of Bais** |
| --- | --- | --- | --- | --- | --- | --- | --- | --- | --- | --- | --- | --- | --- | --- | --- | --- | --- | --- | --- | --- | --- |
| Zhao et al (2023) | Y | Y | Y | Y | Y | Y | Y | Y | Y | Y | Y | Y | N | Y | N | Y | NI | Y | N | NI | 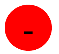 |
| Liu et al (2020) | Y | Y | Y | Y | Y | Y | Y | Y | Y | Y | NI | Y | N | Y | Y | Y | Y | NI | NI | NI | 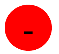 |
| Hui et al (2023) | Y | Y | Y | Y | Y | Y | Y | Y | Y | Y | Y | N | NI | Y | N | Y | NI | N | NI | NI | 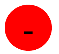 |
| Momenzadeh et al (2022) | Y | Y | Y | Y | Y | Y | Y | Y | Y | Y | Y | Y | N | Y | Y | Y | NI | Y | Y | NI | 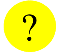 |
| Cai et al (2024) | Y | Y | Y | N | Y | Y | Y | Y | Y | Y | NI | Y | Y | Y | Y | N | NI | NI | N | NI | 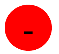 |
| Betzler et al (2023) | Y | Y | Y | Y | Y | Y | Y | Y | Y | Y | Y | Y | Y | Y | Y | Y | Y | Y | Y | Y | 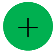 |
| He et al (2024) | Y | Y | Y | Y | Y | Y | Y | Y | Y | Y | Y | N | Y | Y | N | Y | Y | N | Y | NI | 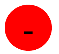 |
| Hosseini Sarkhosh et al | Y | Y | Y | Y | Y | Y | Y | Y | Y | Y | Y | Y | Y | Y | Y | Y | Y | Y | Y | Y | 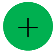 |
| Afrash et al (2022) | N | N | Y | Y | N | N | N | Y | N | N | NI | Y | Y | Y | N | Y | NI | N | Y | NI | 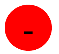 |
| Yang et al (2024) | Y | Y | Y | Y | Y | Y | Y | Y | Y | Y | Y | N | Y | Y | Y | Y | NI | Y | Y | Y | 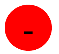 |
| Yin et al (2024) | N | N | Y | Y | Y | N | N | N | Y | N | NI | Y | Y | Y | N | Y | NI | Y | Y | Y | 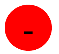 |
| Song et al (2020） | Y | Y | Y | Y | Y | Y | Y | Y | Y | Y | Y | Y | Y | Y | Y | Y | Y | Y | N | N | 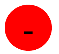 |
| Fan et al (2021) | Y | Y | Y | Y | Y | Y | Y | Y | Y | N | NI | N | Y | Y | N | N | NI | N | N | Y | 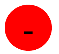 |
| Zhang et al (2022) | Y | Y | Y | Y | N | Y | Y | Y | Y | N | NI | Y | Y | Y | Y | Y | Y | N | Y | NI | 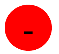 |
| Liu et al (2023) | N | Y | Y | N | Y | Y | Y | Y | Y | N | Y | Y | Y | Y | Y | Y | NI | Y | Y | Y | 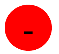 |
| Hosseini Sarkhosh et al (2022) | Y | Y | Y | Y | Y | Y | Y | Y | Y | N | Y | Y | Y | Y | Y | Y | Y | N | Y | Y | 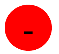 |
| Dong et al (2022) | N | Y | Y | Y | Y | Y | Y | Y | Y | Y | Y | Y | Y | Y | Y | Y | Y | N | N | Y | 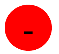 |
| Nicolucci et al (2022) | Y | Y | NI | NI | NI | NI | NI | NI | NI | Y | Y | Y | Y | Y | N | Y | Y | N | Y | NI | 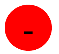 |
| Sabanayagam et al (2023) | Y | Y | Y | Y | Y | Y | Y | Y | Y | Y | Y | N | Y | Y | Y | Y | Y | N | Y | NI | 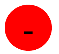 |
| Allen et al (2022) | Y | Y | Y | Y | Y | Y | Y | Y | Y | Y | Y | Y | Y | Y | Y | Y | Y | N | Y | NI | 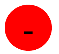 |
| Maniruzzaman et al (2021) | N | NI | NI | NI | N | NI | NI | NI | NI | N | NI | Y | Y | Y | NI | Y | NI | N | N | N | 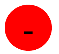 |
| Song et al (2019) | N | Y | Y | Y | Y | Y | Y | Y | Y | Y | Y | N | Y | Y | NI | Y | Y | Y | Y | Y | 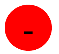 |
| Wang et al (2021) | Y | Y | Y | Y | Y | Y | Y | Y | Y | Y | Y | Y | Y | Y | Y | N | Y | Y | N | Y | 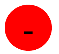 |
| Shi et al (2023) | Y | Y | Y | Y | Y | Y | Y | Y | Y | N | Y | Y | Y | Y | Y | Y | Y | Y | Y | Y | 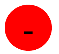 |
| Leung et al (2013) | Y | Y | Y | Y | Y | Y | Y | Y | Y | Y | Y | N | Y | Y | Y | N | Y | N | Y | Y | 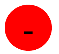 |
| Su et al (2023) | N | Y | Y | Y | Y | N | Y | Y | Y | N | Y | N | N | Y | Y | Y | NI | Y | Y | N | 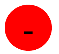 |

Abbreviations: Y, Yes/probably; N, No/probably not; NI, No information;
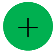
, Low risk;
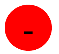
, Hight risk;
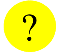
, unclear;

## 1.2 Supplementary Table 3 Pooled AUC of each included study

| **Study** | **Machine Learning** | **Pooled AUC 95% CI** | |
| --- | --- | --- | --- |
|  |  | **Internal validation** | **External validation** |
| Zhao et al (2023) | LR | 0.92(0.871-0.796) | 0.855(0.814-0.895) |
| Liu et al (2020) | BN, BN-wopi, NB, RF, DT | 0.827(0.807-0.847) | - |
| Hui et al (2023) | LASSO | 0.8245(0.75-0.899) | - |
| Momenzadeh et al (2022) | SVC, GBDT, ET, AdaBoost, RF, LR | 0.727(0.721-0.733) | - |
| Cai et al (2024) | DT, RF | 0.801(0.69-0.913) | - |
| Betzler et al (2023) | DL | 0.866(0.859-0.875) | 0.796(0.731-0.862) |
| He et al (2024) | LASSO, GBDT, LR | 0.817(0.77-0.865) | 0.740(0.666-0.814) |
| Hosseini Sarkhosh et al (2023) | LR | 0.755(0.73-0.78) | 0.758(0.730-0.780) |
| Afrash et al (2022) | DT | 0.989(0.988-0.99) | - |
| Yang et al (2024) | DL | 0.910(0.82-0.991) | - |
| Yin et al (2024) | XGB, RF, DT, LR | 0.892(0.821-0.962) | - |
| Song et al（2020） | GBM | 0.83(0.76-0.85) | - |
| Fan et al（2021） | BN, CHAID, D, XF | 0.796(0.707-0.888) | - |
| Zhang et al (2022) | RF, SVM | 0.948(0.942-0.955) | 0.915(0.888-0.942) |
| Liu et al (2023) | CatBoost, GBM, XGBoost, ET, GBDT, RF, LDA, LR, QDA, AdaBoost, NB, KNN, DT, SVM, RC | 0.762(0.729-0.795) | - |
| Hosseini Sarkhosh et al (2022) | DT, SVM, LR, RF, XGBoost | 0.774(0.713-0.843) | - |
| Dong et al（2022） | GBM, XGBoost, AdaBoost, ANN, DT, SVM, LR | 0.769(0.714-0.824) | - |
| Nicolucci et al (2022) | XGBoost | - | 0.898(0.817-0.979) |
| Sabanayagam et al (2023) | LASSO, EN, GBDT | 0.83(0.809-0.851) | - |
| Allen et al（2022） | RF, XGBoost | 0.749(0.74-0.758) | 0.769(0.762-0.776) |
| Maniruzzaman et al (2021） | LDA, SVM-RBF, LR, KNN, NB | 0.84(0.787-0.893) | - |
| Song et al (2019) | GBM | 0.82(0.81-0.83) | 0.710(0.680-0.730) |
| Wang et al (2021) | LR | 0.822(0.805-0.838) | - |
| Shi et al (2023) | RF, SVM, GBDT, Adaboost | 0.874(0.84-0.908) | - |
| Leung et al (2013) | SVM, Cforest | 0.925(0.896-0.954) | - |
| Su et al (2023) | KNN, SVM, LR | 0.885(0.835-0.935) | - |

## Supplementary Figures


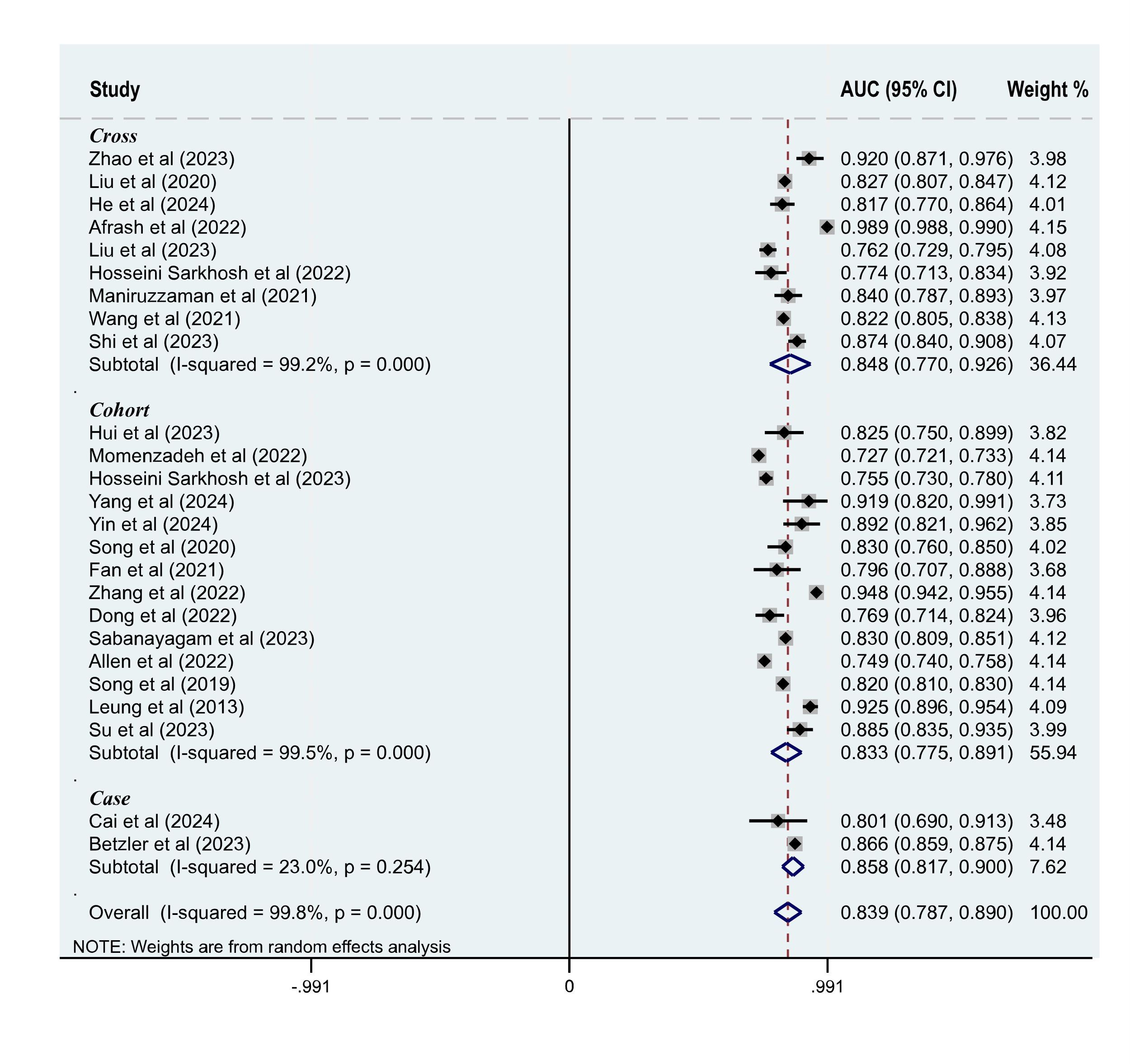


**Supplementary Figure 1** Random effects forest plot of AUC in study type for predicting DKD risk in T2DM using ML.


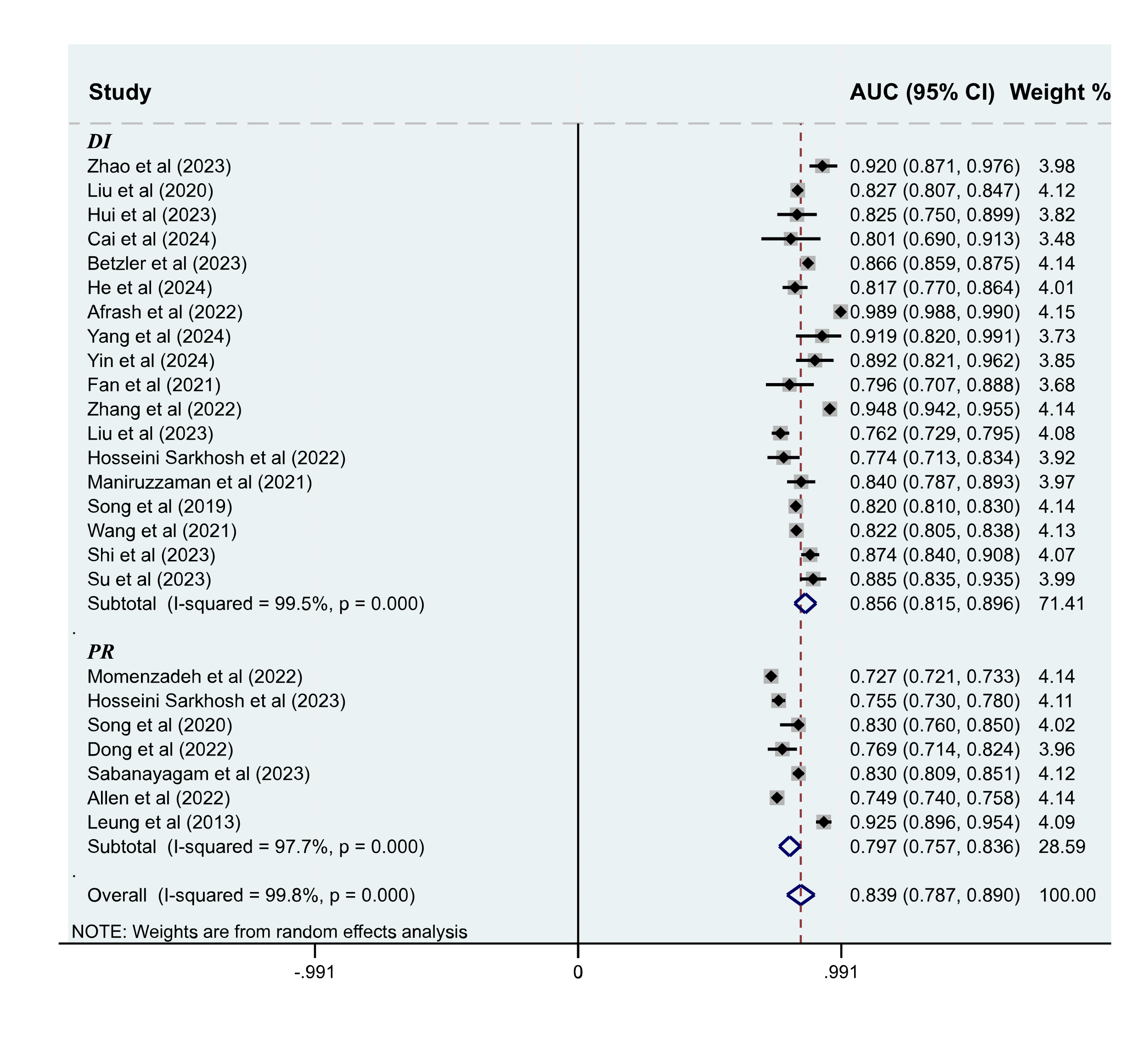


**Supplementary Figure 2** Random effects forest plot of AUC in predictive model type for predicting DKD risk in T2DM using ML.


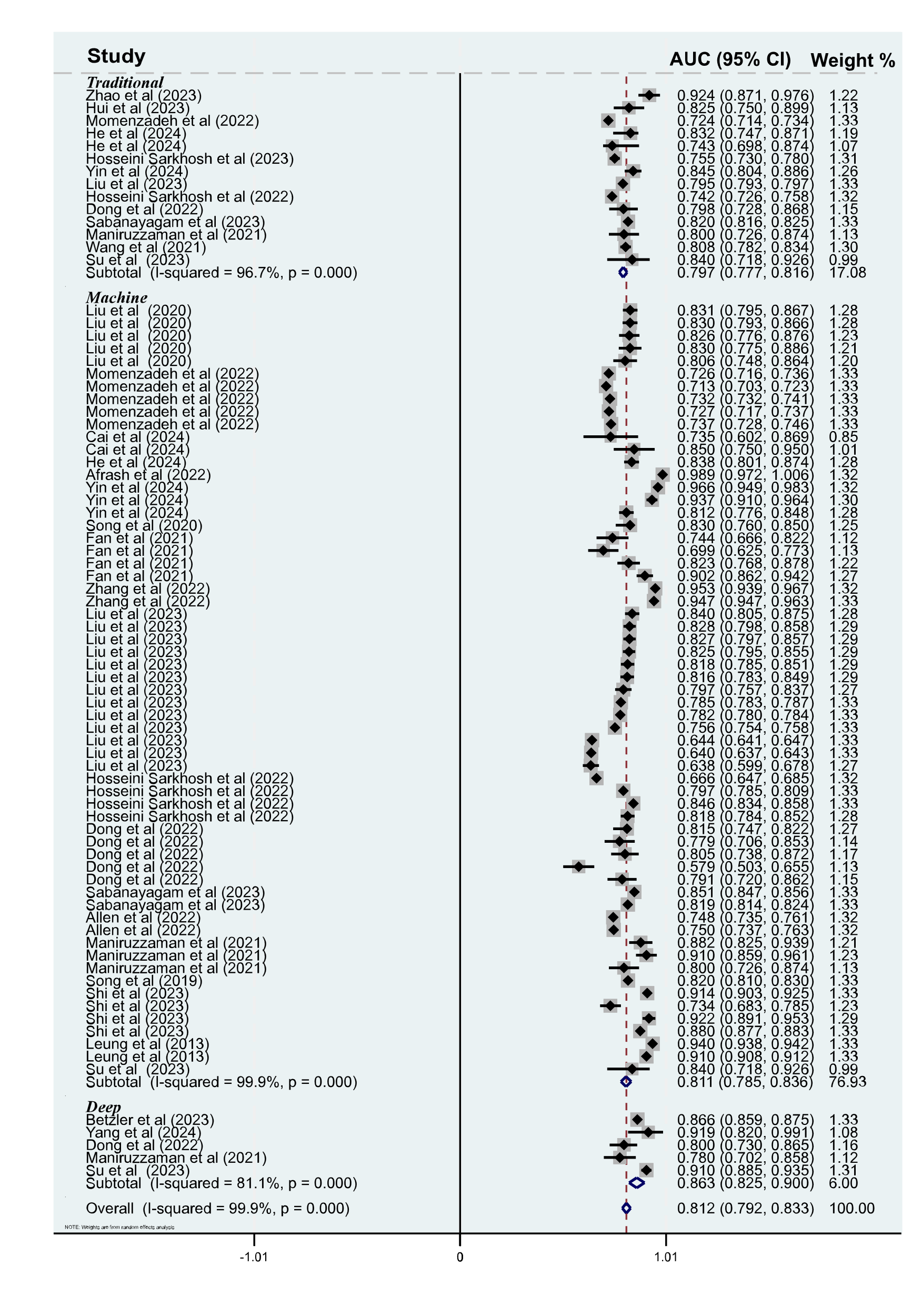


**Supplementary Figure 3** Random effects forest plot of AUC in ML model type


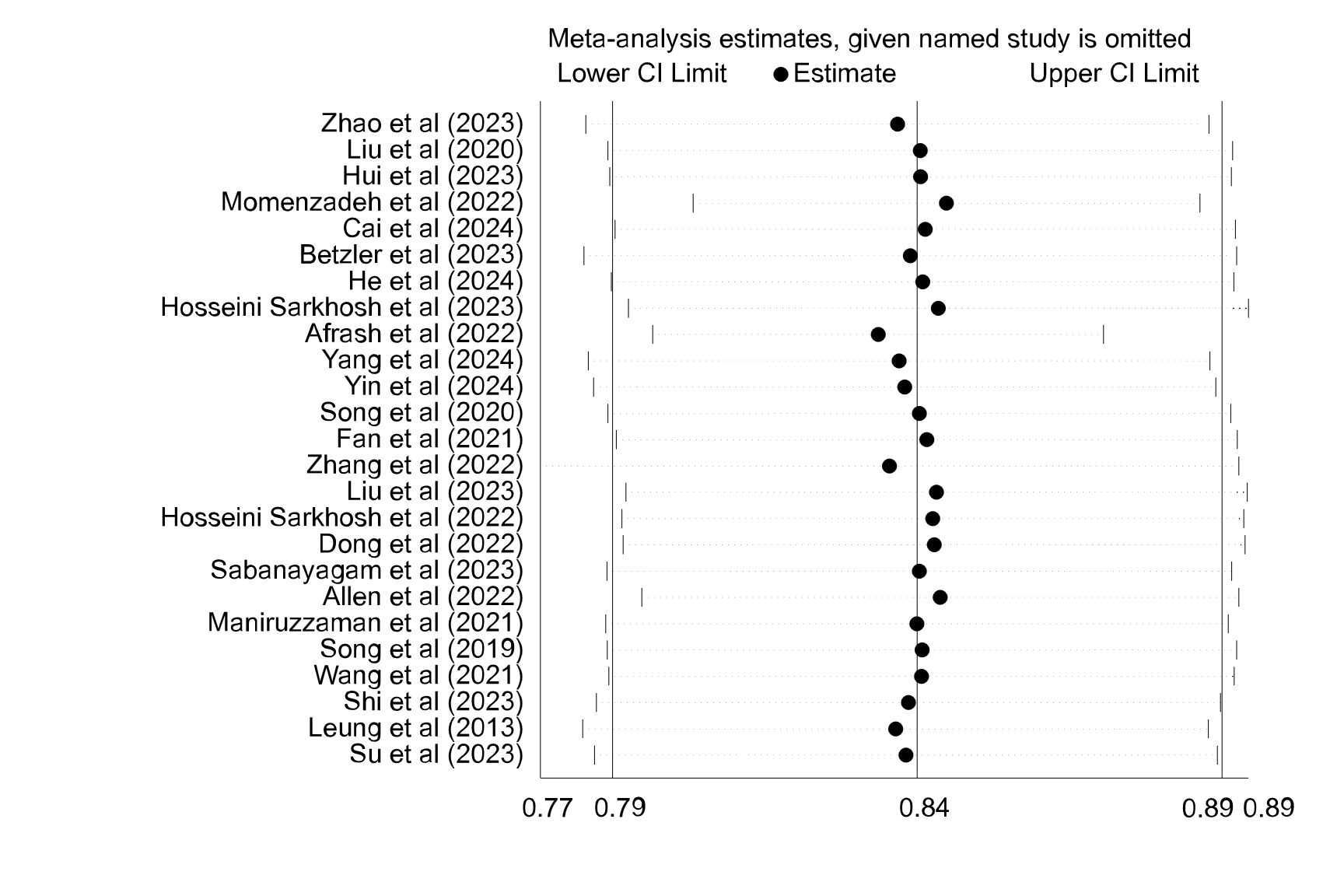


**Supplementary Figure 4** The sensitivity analysis
